# Supplementary material for: A zero-agnostic model for copy number evolution in cancer
Source: PLoS Comput Biol. 2023 Nov 9;19(11):e1011590. doi: 10.1371/journal.pcbi.1011590 (PMC10662746; doi:10.1371/journal.pcbi.1011590)
Supplement: S1 Text — Supplementary text file (PDF) containing supplementary methods, results, and proofs. (PDF) [file pcbi.1011590.s001.pdf]

S1 Text: A zero-agnostic model for copy number evolution in cancer

Henri Schmidt[1], Palash Sashittal[1], and Benjamin J. Raphael[1,*]

[1]Department of Computer Science, Princeton University, NJ, USA
*Correspondence: braphael@princeton.edu

A Supplementary Methods

A.1 Large parsimony details

Our tree-search algorithm starts with an initial set of candidate trees $S = (\mathcal{T}_1, \ldots, \mathcal{T}_k)$ and iteratively improves upon the trees by stochastic perturbations followed by a hill-climbing procedure . Specifically, at each iteration we select a candidate tree uniformly at random and perturb the tree using a random number r of nearest neighbor interchange (NNI) operations. With our perturbed candidate tree, we then perform local optimization using hill climbing to minimize the cost of the tree, where we use our small parsimony algorithm to efficiently evaluate the cost of each candidate topology. Once the hill-climbing procedure reaches a local minimum, we complete the iteration and update the candidate tree set if an improvement was found. The algorithm terminates after no improvement is found for a fixed number I of iterations.

For all experiments and analysis in this paper, the number of iterations prior to termination was set to $I = 150$. The number of random NNIs to perturb the candidate tree is selected uniformly at random from the discrete interval $\{0, 1, \ldots, \lfloor 2.5n \rfloor\}$ at every iteration. Our candidate tree set was generated by performing neighbor joining on the boundary insensitive distances and then randomly perturbing the the neighbor joining tree.

A.2 Simulation details

We used a modified version of CONET's [1] copy number phylogeny simulator. Specifically, we found that CONET's simulation of tree structure was non-standard and opted to use a forward-birth death model [2] to simulate our topology. Once the tree structure was generated, we then used CONET's simulator to sample copy number events on each vertex. We then took our event labeled copy number phylogeny and sampled the ground truth copy number states on the leaves of the phylogeny to obtain our copy number profiles.

To generate the tree topology, we used Cassiopeia's [3] implementation of a forward-birth death model. We performed simulations for $n = 100, 150, 200, 250, 600$ leaves with a fitness parameter of 1.3 and an initial birth scale of 0.5. We drew the birth-death waiting times from an exponential distribution. With the topology, we randomly sampled events on each vertex using CONET with $l = 1000, 2000, 3000, 4000$ loci. We performed each simulation with parameters (n, l) a total of 7 times with unique random seeds $s = 0, 1, 2, 3, 4, 5, 6$. In total, there were 140 randomly simulated instances.

A.3 Clonal concordance analysis

To analyze the concordance of the inferred phylogenetic trees with clonal information, we measured the minimum number of evolutionary events required to explain the clones. Specifically, for each sample clones were identified by clustering the GC-corrected read count profiles embedded using UMAP [4, 5]. The clone labels were then attached to the leaves of the inferred phylogenetic trees. With this clone labeled phylogenetic tree, we solved the small parsimony problem under the Wagner [6] model to obtain a parsimony score, p, which we call the *clonal discordance score*. This clonal discordance score is the minimum number of clonal transitions required to explain the cells of the phylogeny.

To compare across different phylogeny sizes, we computed the relative clonal discordance score between the *Lazac* and Sitka phylogenies as

$$r = \frac{p_2 - p_1}{p_1 + p_2},$$

where p_1, p_2 are the clonal discordance scores of the *Lazac* and Sitka phylogenies respectively. In particular, a positive score indicates that the *Lazac* phylogeny is more concordant with the clones while a negative score indicates that the Sitka phylogeny is more concordant with the clones.

A.4 Permutation test for analysis of SNV support

In this section we provide details about how subtrees of the phylogenies are identified for the analysis and the permutation test used for investigate if the subtrees are supported by the SNVs.

First, we describe how we identify subtrees in the phylogeny to analyze. Our goal is to identify subtrees that have enough cells so that pooling the reads from the cells and finding SNVs is feasible. However, if the number of cells is too large, the permutation test will not yield a significantly low p-value. To that end, we perform a breadth-first traversal of the nodes of the tree (starting from the root) to identify the desired subtrees. At each iteration, we compute the number of cells in the subtree rooted at the node (i.e. the number of leaves in the subtree). We select the subtree if (1) the number of cells in the subtree is more than 10% of the total number of cells (2) the number of cells in the subtree is less than 25% of the total number of cells and (3) the subtree is not contained in any of the subtrees selected in previous iterations.

Now, we provide details about the permutation test for a given subtree. We say that an SNV supports a subtree of a phylogeny if all the cells that yield a read harboring the SNV are contained in the subtree. We randomly permute the cell labels 500 times and count the number of SNVs supporting a given subtree. The p-value is empirically estimated by the ratio of the number of instances in which more SNVs support the subtree than with the original unpermuted cell labels with the total number of permutations tested (which is 500 in our study).

A.5 Comparison to simulated trees

We assess the accuracy of the inferred trees compared to the ground truth simulated trees by employing two distinct tree dissimilarity metrics. These metrics are implemented in the TreeCmp tool [7] and the comparisons are done in a similar manner to the comparisons in our *Startle* [8] paper. Our metrics take a ground truth tree, $\mathcal{T}^*$, and an inferred tree, $\mathcal{T}$, both in Newick format [9].

The Robinson-Foulds (RF) distance, $d_{\text{RF}}(\mathcal{T}, \mathcal{T}^*)$, is a tree distance metric based on the induced bi-partitions in the input trees [10, 11]. Each edge $e \in \mathcal{T}$ is associated with a bi-partition $B_e := (X, \bar{X})$ of its leaves, using the equivalence relation $x \sim y$ if x is connected to y in $\mathcal{T}_{-e}$, the forest formed by removing edge e. The set of bi-partitions for a tree $\mathcal{T}$ is $\text{Bip}(\mathcal{T}) = \{B_e : e \in E(\mathcal{T})\}$. The RF distance is then:

$$d_{\text{RF}}(\mathcal{T}, \mathcal{T}*) = |\text{Bip}(\mathcal{T}) \triangle \text{Bip}(\mathcal{T}^*)|.$$

Similarly, the quartet distance, $d_Q(\mathcal{T}, \mathcal{T}^*)$, is a tree distance metric based on the induced quartets in the input trees [10, 11]. We define the set of quartets $Q(\mathcal{T})$ as the set of all consistent 4-leaf sub-trees with the unrooted topology of $\mathcal{T}$. Then,

$$d_Q(\mathcal{T}, \mathcal{T}*) = |Q(\mathcal{T}) \vartriangle Q(\mathcal{T}^*)|.$$

Finally, we used normalized versions of both d_{RF} and d_Q to enable comparison across different parameter settings. This normalization is implemented in TreeCmp [7] and described in their paper.

B Supplementary Results

B.1 ZCNT small parsimony: dropping the integrality condition

Let Q be the delta matrix obtained by applying the delta transformation to each row of the copy number matrix M (i.e. $q_{ij} = \Delta(m_i)_j$). Using the formulation of the ZCNT small parsimony problem as stated in (Problem 3), we can write the objective as the following mathematical program.

$$\begin{aligned} \min_{\ell} \quad & \sum_{(u,v)\in E(\mathcal{T})} \frac{1}{2}\|\ell(u) - \ell(v)\|_1 \\ \text{s.t.} \quad & l(u) \in \mathbb{Z}^m \quad \text{for all } u \in V(\mathcal{T}), \\ & \sum_{j=1}^{m} l(u)_j = 0 \quad \text{for all } u \in V(\mathcal{T}), \\ & l(\pi(i))_j = Q_{ij} \quad \text{for all } i \in [n], j \in [m]. \end{aligned}$$

Notice that we can rewrite the optimization objective as a linear function subject to additional constraints. Specifically, $\|\ell(u)-\ell(u)\|_1 = \sum_{j=1}^{m}(x^+_{uvj}-x^-_{uvj})$ when $x^+_{uvj} = \max\{\ell(u)_j, \ell(v)_j\}$ and $x^-_{uvj} = \min\{\ell(u)_j, \ell(v)_j\}$. And we can set x^+_{uvj} using the two linear constraints $x^+_{uvj} \geq \ell(u)_j$ and $x^+_{uvj} \geq \ell(v)_j$; a similar procedure works for x^-_{uvj}. Then, by dropping the integrality condition $\ell(u) \in \mathbb{Z}^m$, we obtain the following equivalent linear program.

$$\begin{aligned} \min_{x,\ell} \quad & \frac{1}{2} \sum_{(u,v)\in E(\mathcal{T})} \sum_{j=1}^{m} (x^+_{uvj} - x^-_{uvj}) \\ \text{s.t.} \quad & \sum_{j=1}^{m} \ell(u)_j = 0 && \text{for all } u \in V(\mathcal{T}), \\ & \ell(\pi(i))_j = Q_{ij} && \text{for all } i \in [n] \text{ and } j \in [m], \\ & x^+_{uvj} \geq \ell(u)_j \text{ and } x^+_{uvj} \geq \ell(v)_j && \text{for all } (u,v) \in E(\mathcal{T}) \text{ and } j \in [m], \\ & x^-_{uvj} \leq \ell(u)_j \text{ and } x^-_{uvj} \leq \ell(v)_j && \text{for all } (u,v) \in E(\mathcal{T}) \text{ and } j \in [m]. \end{aligned}$$

Since we can solve linear programs in (weakly) polynomial time, this proves the following theorem.

Theorem 1. *The ZCNT small parsimony problem can be solved in (weakly) polynomial time when the constraint that $\ell(u) \in \mathbb{Z}^m$ is relaxed to $\ell(u) \in \mathbb{R}^m$ using a linear program with $O(mn)$ variables and $O(mn)$ constraints.*

B.2 ZCNT small parsimony: dropping the balancing condition

When we drop the balancing condition, our problem becomes equivalent to the fixed topology rectilinear Steiner tree problem [12] on the delta profiles where the ancestral nodes lie in $\mathbb{Z}^m$. While there are several algorithms for the unrooted variant of this problem when Steiner vertices are in $\mathbb{R}^m$ [13, 12, 14], our problem is different in that i) it assumes a rooted topology ii) the Steiner vertices are required to lie in $\mathbb{Z}^m$. In this section, we present and prove the correctness of a linear time dynamic programming algorithm that solves the ZCNT small parsimony problem when the balancing condition is dropped.

We first observe that it suffices to analyze each locus independently. Let Q be the delta matrix obtained by applying the delta transformation to each row of the copy number matrix M (i.e. $q_{ij} = \Delta(m_i)_j$). Let ℓ_j minimize the quantity $\sum_{(u,v)\in E(\mathcal{T})} |\ell_j(u) - \ell_j(v)|$ and agree with the delta matrix Q on the leaves; that is, $\ell_j(\pi(i)) = q_{ij}$ for all cells $i \in [n]$. Then, the labeling defined as $\ell(u) = (\ell_1(u), \ldots, \ell_m(u))$ minimizes

Algorithm 1 ZCNT small parsimony without the balancing condition

Require: A binary tree $\mathcal{T}$ rooted at vertex v, a delta matrix Q, an assignment π of cells to leaves, and a locus j.

Output: A minimizer of the cost $J(\ell_j, \mathcal{T})$ for locus j that agrees with Q on the leaves of $\mathcal{T}$.

```
if v is a leaf in T then
    set ℓ_j(v) ← [q_{π(v),j}, q_{π(v),j}]
    return
end if

get w, z ← children(v)
recurse at nodes w and z
set l_j(v) ← Sank(l_j(w), l_j(v))
```

$J(\ell, \mathcal{T})$. To see this, note that

$$\begin{aligned}
\min_{\hat{\ell}} J(\hat{\ell}, \mathcal{T}) &= \min_{\hat{\ell}} \sum_{(u,v)\in E(\mathcal{T})} \frac{1}{2} \left\|\hat{\ell}(u) - \hat{\ell}(v)\right\|_1 \\
&= \min_{\hat{\ell}} \left(\frac{1}{2} \sum_{j=1}^{m} \sum_{(u,v)\in E(\mathcal{T})} \left|\hat{\ell}(u)_j - \hat{\ell}(v)_j\right| \right) \\
&= \frac{1}{2} \sum_{j=1}^{m} \min_{\hat{\ell}_j} \left(\sum_{(u,v)\in E(\mathcal{T})} \left|\hat{\ell}_j(u) - \hat{\ell}_j(v)\right| \right) \\
&= J(\ell, \mathcal{T}),
\end{aligned}$$

where the second equality follows from the definition of the ℓ_1 norm and the second equality from the separability of objective. Thus, we can compute the cost of the optimal labeling ℓ_j for each locus independently and sum them together to obtain the entire cost.

We introduce the quantity $c(\mathcal{T}; x)$ as the cost $J(\ell, \mathcal{T})$ of the optimal labeling ℓ of $\mathcal{T}$ that agrees with Q on the leaves of $\mathcal{T}$ and has root label x. Our algorithm relies on the following easy to compute function on discrete intervals denoted $[a, b] = \{a, a+1, \ldots, b-1, b\}$:

$$\text{Sank}([a,b],[c,d]) = \begin{cases} [a,b] \cap [c,d] & \text{if } [a,b] \cap [c,d] \neq \{\}, \\ [b,c] & \text{if } b \leq c, \\ [d,a] & \text{otherwise if } d \leq a. \end{cases}$$

Our algorithm then applies the Sank$(\cdot,\cdot)$ function in a top-down recursive fashion, to compute *the interval* of optimal root labelings for each node. Though this procedure is quite natural, its proof of correctness is not immediately obvious and relies on a technical (Lemma 3).

Theorem 2. *(Algorithm 1) solves the ZCNT small parsimony problem in $O(n)$ time for a single locus when the balancing condition is dropped.*

Proof. Follows from induction on the size of the tree using (Lemma 3). ■

To prove the correctness of our procedure, we introduce the function $\text{dist}(x, [a, b])$ defined as the distance from x to the discrete interval $[a, b]$:

$$\text{dist}(x, [a, b]) = \begin{cases} 0 & \text{if } x \in [a, b] \\ \min\{|x - a|, |x - b|\} & \text{otherwise.} \end{cases}$$

The correctness of our algorithm relies on two technical lemmas whose proofs are in (Section C).

Lemma 1. *If $a \leq b < c \leq d$ are integers, then for all integers x the following inequality holds:*

$$\min_{y,z\in\mathbb{Z}} (dist(y, [a, b]) + dist(z, [c, d]) + |x - y| + |x - z|) \geq (c - b) + dist(x, [b, c]).$$

Lemma 2. *If $a \leq b$, $c \leq d$, $a \leq c$, and $b \geq c$ are integers, then for all integers x the following inequality holds:*

$$\min_{y,z\in\mathbb{Z}} (dist(y, [a, b]) + dist(z, [c, d]) + |x - y| + |x - z|) \geq dist(x, [c, b]).$$

Then, the correctness of our algorithm follows from induction using the following lemma.

Lemma 3. *Let $\mathcal{T}$ be a tree whose root vertex v has two children v_1 and v_2. Let $\mathcal{T}_{v_1}$ and $\mathcal{T}_{v_2}$ denote the sub-trees rooted at v_1 and v_2 respectively. Then, suppose that*

$$c(\mathcal{T}_{v_1}; x) \geq c(\mathcal{T}_{v_1}) + dist(x, [a, b])$$
$$\text{and } c(\mathcal{T}_{v_2}; x) \geq c(\mathcal{T}_{v_2}) + dist(x, [c, d])$$

where $[a, b]$ and $[c, d]$ are the set of optimal root labelings for $\mathcal{T}_{v_1}$ and $\mathcal{T}_{v_2}$. Then, $[e, f] = \textsc{Sank}([a, b], [c, d])$ is the optimal set of root labelings for $\mathcal{T}$ and

$$c(\mathcal{T}; x) \geq c(\mathcal{T}_{v_1}) + c(\mathcal{T}_{v_2}) + dist(x; [e, f]) + \mathbb{1}[b < c](c - b).$$

Proof. Without loss of generality we can assume that $a \leq c$. Otherwise, we can swap the names of vertices v_1 and v_2. Let x be the labeling of the root of $\mathcal{T}$. Then,

$$\begin{aligned} c(\mathcal{T}; x) &= \min_{y,z\in\mathbb{Z}} \left[c(\mathcal{T}_{v_1}; y) + c(\mathcal{T}_{v_2}; z) + |x - y| + |x - z|\right] \\ &\geq \min_{y,z\in\mathbb{Z}} [\, c(\mathcal{T}_{v_1}) + \text{dist}(y; [a, b]) + c(\mathcal{T}_{v_2}) + \text{dist}(z; [c, d]) + |x - y| + |x - z|] \end{aligned}$$

where the equality follows from the definition of $c(\cdot;\cdot)$ and the fact that we are using the ℓ_1 distance. And the inequality follows from the theorem assumptions about $c(\mathcal{T}_{v_1}; y)$ and $c(\mathcal{T}_{v_2}; z)$.

We now consider the two cases of $\textsc{Sank}([a, b], [c, d])$ separately.

Case 1: $b < c$. In this case, we know from definition that $\textsc{Sank}([a, b], [c, d]) = [b, c]$. We want to show that

$$\begin{aligned} &\min_{y,z\in\mathbb{Z}} (\text{dist}(y, [a, b]) + \text{dist}(z, [c, d]) + |x - y| + |x - z|) \\ &\qquad\qquad \geq (c - b) + \text{dist}(x, [b, c]). \end{aligned}$$

This will prove the desired inequality of the theorem. Then, to see that $[b, c]$ is the optimal labeling of the root it is enough to observe that the inequality is realized when $x \in [b, c]$. As the proof of this inequality

is rather technical and unenlightening, it is summarized in in (Lemma 1) and proven in Supplementary Proofs.

Case 2: $\textsc{Sank}([a,b],[c,d]) = [c,b]$ and $c \leq b$. In this case, we want to show that

$$\min_{y,z\in\mathbb{Z}} (\text{dist}(y,[a,b]) + \text{dist}(z,[c,d]) + |x-y| + |x-z|) \\ \geq \text{dist}(x,[b,c]).$$

Which will again prove the desired inequality of the theorem. Then, to see that $[c,d]$ is the optimal labeling of the root it is enough to observe that the inequality is realized when $x \in [c,d]$. The proof of this inequality is given in (Lemma 2). ■

B.3 ZCNT small parsimony: a relabeling strategy and approximation algorithm

In this section, we provide complete details for the relabeling strategy and approximation algorithms described in (Section 2.4.2). First, notice that for any labeling ℓ that agrees with the copy number matrix M on the leaves of $\mathcal{T}$ (i.e. $\ell(\pi(i)) = \Delta(M_i)$ for all cells $i \in [n]$), we have $\text{disc}(\ell(u)) = 0$ for all leaves in $u \in L(\mathcal{T})$. Given any such labeling, we first show that we can alter the labeling to decrease the total vertex discrepancy with only a small penalty to the parsimony objective.

Lemma 4. *Let $\ell : V(\mathcal{T}) \to \mathbb{Z}^m$ be any labeling of a binary tree $\mathcal{T}$ such that $\ell(\pi(i)) = \Delta(M_i)$ for all cells $i \in [n]$. Then, if $disc(\ell(u)) \neq 0$ for any vertex $u \in V(\mathcal{T})$, we can construct a new labeling ℓ' such that*

$$\sum_{u\in V(\mathcal{T})} |disc(\ell'(u))| = \sum_{u\in V(\mathcal{T})} |disc(\ell(u))| - 1 \quad \text{and} \quad J(\mathcal{T},\ell') \leq 1 + J(\mathcal{T},\ell).$$

Proof. Let u be a deepest vertex (i.e. a vertex such that the distance from the root of $\mathcal{T}$ is maximal) such that $\text{disc}(\ell(u)) \neq 0$. Assume that $\text{disc}(\ell(u)) > 0$. Since $\mathcal{T}$ is a binary tree and the discrepancy of all leaves is zero, u has children w, v such that $\text{disc}(\ell(w)) = \text{disc}(\ell(v)) = 0$. Then, since $\text{disc}(\ell(u)) > \text{disc}(\ell(w))$ there exists a coordinate $j \in [m]$ such that $\ell(u)_j > \ell(w)_j$ as otherwise,

$$\text{disc}(\ell(u)) = \sum_{j=1}^{m} \ell(u)_j \leq \sum_{j=1}^{m} \ell(w)_j = \text{disc}(\ell(w)),$$

a contradiction. We create a new labeling ℓ' that is identical to ℓ except that $\ell'(u)_j = \ell(u)_j - 1$. Then, since $\ell(u)_j > \ell(w)_j$, the distance between u and w has decreased by one, the distance from u to v has increased by at most one, and the distance to w's parent, if it exists, has increased by at most one. As no other distances have been altered, ℓ' is a new labeling such that $J(\mathcal{T},\ell') \leq J(\mathcal{T},\ell) + 1$. Further, the total vertex discrepancy has been decreased by one.

The case where $\text{disc}(\ell(u)) < 0$ follows by a symmetric argument by reversing the sign of the inequalities and updating the labeling as $\ell'(u)_j = \ell(u)_j + 1$. This completes the proof. ■

Notice that by repeating the relabeling strategy given by (Lemma 4) until the discrepancy of every vertex is zero, we can make any labeling satisfy the balancing condition. Further, it is possible to do this in $O(mn)$ time by performing the relabelings in a bottom-up tree traversal. These observations prove the following corollary.

Corollary 1. *Let $\ell : V(\mathcal{T}) \to \mathbb{Z}^m$ be any labeling of a binary tree $\mathcal{T}$ such that $\ell(\pi(i)) = \Delta(M_i)$ for all cells $i \in [n]$. Then, we can construct a new labeling ℓ' satisfying the balancing condition such that:*

$$J(\mathcal{T}, \ell') \leq J(\mathcal{T}, \ell) + \sum_{u \in V(\mathcal{T})} |disc(\ell(u))|.$$

Further, we can compute the labeling ℓ' in $O(mn)$ time.

We then prove that this does in fact give us a 2-approximation algorithm, by showing that the total cost $J(\mathcal{T}, \ell)$ is at lower bounded by the total vertex discrepancy.

Lemma 5. *Let $\ell : V(\mathcal{T}) \to \mathbb{Z}^m$ be any labeling of a binary tree $\mathcal{T}$ such that $\ell(\pi(i)) = \Delta(M_i)$ for all cells $i \in [n]$. Then*

$$J(\mathcal{T}, \ell) \geq \sum_{u \in V(\mathcal{T})} |disc(\ell(u))|.$$

Proof. We have the following set of inequalities

$$\begin{aligned}
J(\mathcal{T}, \ell) &= \sum_{(u,v) \in E(\mathcal{T})} \|\ell(u) - \ell(v)\|_1 \\
&\geq \sum_{(u,v) \in E(\mathcal{T})} |\,\text{disc}(\ell(u)) - \text{disc}(\ell(v))\,| \\
&\geq \sum_{(u,v) \in E(\mathcal{T})} |\text{disc}(\ell(u))| - |\text{disc}(\ell(v))| \\
&\geq 2 \cdot \sum_{u \in V(\mathcal{T})} |\text{disc}(\ell(u))| - \sum_{u \in V(\mathcal{T})} |\text{disc}(\ell(u))| \\
&= \sum_{u \in V(\mathcal{T})} |\text{disc}(\ell(u))|
\end{aligned}$$

where the first inequality follows from the triangle inequality: $\|x\|_1 \geq |\sum_{j=1}^{m} x_j|$. The second inequality follows from the relation $|a - b| \geq |a| - |b|$. And the final inequality from the fact that each internal vertex u appears twice as the parent of children, at most once as a child, and each leaf labeling has discrepancy zero. Said another way, each internal vertex u appears twice in the left hand side of the sum and at most once in the right hand side of the sum, while the leaves do not contribute at all to the sum. ∎

C Supplementary Proofs

Theorem 3. *Let $c_{s,t,b}$ be a zero-agnostic copy number event and $\delta_{s,t,b}$ be a delta event. Then,*

$$p' = c_{s,t,b}(p) \quad \text{if and only if} \quad \Delta(p') = \delta_{s,t,b}(\Delta(p)).$$

Proof. ($\Rightarrow$) Let p' be the result of applying the zero-agnostic copy number event $c_{s,t,b}$ to the profile p. Then, if $i, i-1 \in \{s, \dots, t\}$:

$$\Delta(p')_i = (p_i + b) - (p_{i-1} + b) = p_i - p_{i-1} = \Delta(p)_i.$$

Similarly, if $i, i-1 \notin \{s, \dots, t\}$

$$\Delta(p')_i = p_i - p_{i-1} = \Delta(p)_i.$$

The remaining two cases occur when either $i = s$ or $i - 1 = t$.

$$\begin{aligned} \Delta(p')_i &= (p_i + b) - p_{i-1} = \Delta(p)_i + b && \text{if} \quad i = s, \\ \Delta(p')_i &= p_i - (p_{i-1} + b) = \Delta(p)_i - b && \text{if} \quad i - 1 = t \end{aligned}$$

Thus, $\Delta(p')$ is the result of applying the delta event δ to the profile $\Delta(p)$.

($\Leftarrow$) This case is handled symmetrically. ∎

Proposition 1. *$d'(q, q')$ is a distance metric. Further,*

$$d'(q, q') = d'(q - q', 0) = d'(q' - q, 0).$$

Proof. To see that $d'(\cdot, \cdot)$ satisfies the triangle inequality, it suffices to observe that the composition of delta sequences taking q to r and r to q' transforms q to q'. It is clearly reflexive since no delta event needs to be applied to map q to itself.

To see symmetry and the above equality, we observe that a stronger property is satisfied. Let

$$\gamma(D)_j := \sum_{i=1}^{n} (b_i * \mathbb{1}[j = s_i] - b_i * \mathbb{1}[j = t_i])$$

be the net change of coordinate j induced by the delta sequence $D = (\delta_1, \dots, \delta_n)$ where $\delta_i = (s_i, t_i, b_i)$. Then, D takes q to q' if and only if $\gamma(D)_j = q'_j - q_j$ for all $j \in \{1, \dots, m\}$. This follows from the definition of our delta event and the fact that applying D to q results in a profile q' defined by its entries as $q'_j = q_j + \gamma(D)_j$. ∎

Proposition 2. *For a vector $p' = \Delta(p)$, the sum of the magnitudes of the positive entries equals the sum of the magnitudes of the negative entries. That is,*

$$\sum_{p'_i > 0} |p'_i| = \sum_{p'_i < 0} |p'_i|. \tag{1}$$

Proof. We first notice that $\sum_{i=0}^{n+1} \Delta(p)_i$ expands to a telescoping sum after applying our definition of a delta profile. Specifically,

$$\begin{aligned} \sum_{i=0}^{n+1} \Delta(p)_i &= (p_0 - 2) + (2 - p_n) + \sum_{i=1}^{n} (p_i - p_{i-1}) \\ &= (p_0 - p_n) + (p_n - p_0) = 0. \end{aligned}$$

Then, we rewrite the left hand side of the sum

$$\begin{aligned}\sum_{i=0}^{n+1}\Delta(p)_i &= \sum_{\Delta(p)_i>0}\Delta(p)_i + \sum_{\Delta(p)_i<0}\Delta(p)_i\\ &= \sum_{\Delta(p)_i>0}\Delta(p)_i - \sum_{\Delta(p)_i<0}-\Delta(p)_i\\ &= \sum_{\Delta(p)_i>0}|\Delta(p)_i| - \sum_{\Delta(p)_i<0}|\Delta(p)_i|,\end{aligned}$$

where the last equality follows from the definition of absolute value. ■

Proposition 3. *The delta map $\Delta : \mathbb{Z}^n \longrightarrow \mathcal{D}_{n+1}$ is invertible.*

Proof. By (Proposition 2) the range of the map Δ lies in the space of vectors in $\mathbb{Z}^{n+1}$ satisfying the balance condition. Thus, it suffices to show that Δ is injective and can reach all vectors (i.e. is surjective) in this space.

To see that the map is injective, let $x, y \in \mathbb{Z}^n$ be two distinct vectors. Then, define i as the first coordinate in which these vectors differ. Since i is the first coordinate in which these vectors differ, $x_{i-1} = y_{i-1}$ but $x_i \neq y_i$. Thus,

$$\Delta(x)_i = x_i - x_{i-1} \neq y_i - y_{i-1} = \Delta(y)_i,$$

proving that Δ is injective.

To see the map is surjective, let $y \in \mathbb{Z}^{n+1}$ be a vector satisfying the balance condition. Define a vector $x \in \mathbb{Z}^n$ such that $x_0 = y_0 + 2$, and $x_i = y_i + x_{i-1}$ for $i \in \{1, \ldots, n\}$. Then, $\Delta(x)$ agrees with y on the first n coordinates, but since y and $\Delta(x)$ both satisfy the balance condition by (Proposition 2), their last coordinate is also determined, proving that $\Delta(x) = y$. ■

Corollary 2. *Given two copy number profiles p and p' in $\mathbb{Z}^n$, both p and p' minimize the median distance $d(r, p) + d(r, p')$ over all choices of copy number profiles r. Thus,*

$$\min_{r\in\mathbb{Z}^n}\{d(r, p) + d(r, p')\} = d(p, p').$$

Proof. By the triangle inequality, $d(r, p) + d(r, p') \geq d(p, p')$ for all profiles r. Setting $r = p$ or $r = p'$ achieves equality, proving that setting r to either p or p' minimizes the expression $d(r, p) + d(r, p')$. ■

Lemma 1. *If $a \leq b < c \leq d$ are integers, then for all integers x the following inequality holds:*

$$\min_{y,z\in\mathbb{Z}}\left(dist(y, [a, b]) + dist(z, [c, d]) + |x - y| + |x - z|\right) \geq (c - b) + dist(x, [b, c]).$$

Proof. The statement follows from a case analysis on the locations of the variables we are optimizing over: y, z. However, before any case analysis, we prove that

$$\text{dist}(x, [a, b]) + \text{dist}(x, [c, d]) \geq \text{dist}(x, [b, c]) + (c - b). \tag{2}$$

To see this, we perform a case analysis on x. If $x \leq b$ we have

$$\text{dist}(x, [c, d]) = \text{dist}(x, [b, c]) + (c - b),$$

since x is to the left of the interval $[b, c]$ which is to the left of the interval $[c, d]$. If $x \geq c$ we have

$$\text{dist}(x, [a, b]) = \text{dist}(x, [b, c]) + (c - b),$$

since x is to the right of the interval $[b, c]$ which is to the right of the interval $[a, b]$. And finally, if $x \in [b, c]$ then,

$$\text{dist}(x, [a, b]) + \text{dist}(x, [c, d]) = (c - b) = \text{dist}(x, [b, c]) + (b - c),$$

since $\text{dist}(x, [b, c]) = 0$.

Case 1: $y \in [a, b]$ and $z \in [c, d]$

In this case, $\text{dist}(y, [a, b]) + \text{dist}(z, [c, d]) = 0$, so

$$\begin{aligned}
&\min_{\substack{y\in[a,b]\\ z\in[c,d]}} (\text{dist}(y, [a, b]) + \text{dist}(z, [c, d]) + |x - y| + |x - z|) \\
&= \min_{\substack{y\in[a,b]\\ z\in[c,d]}} (|x - y| + |x - z|) \\
&= \text{dist}(x, [a, b]) + \text{dist}(x, [c, d]) \\
&\geq (c - b) + \text{dist}(x, [b, c]),
\end{aligned}$$

where the second equality follows from the fact that $\text{dist}(x, [a, b]) = \min_{y\in[a,b]} |x - y|$ and the inequality follows from (2).

Case 2: $y \in [a, b]$ and $z \notin [c, d]$ OR $y \notin [a, b]$ and $z \in [c, d]$

Since the two cases are symmetric, we only need to consider the former situation where $y \in [a, b]$ and $z \notin [c, d]$. In this case,

$$\begin{aligned}
&\min_{\substack{y\in[a,b]\\ z\notin[c,d]}} (\text{dist}(y, [a, b]) + \text{dist}(z, [c, d]) + |x - y| + |x - z|) \\
&= \min_{\substack{y\in[a,b]\\ z\notin[c,d]}} (\text{dist}(z, [c, d]) + |x - y| + |x - z|) \\
&= \text{dist}(x, [a, b]) + \min_{z\notin[c,d]} (\text{dist}(z, [c, d]) + |x - z|) \\
&= \text{dist}(x, [a, b]) + \min_{z\notin[c,d]} (\min\{|z - c|, |z - d|\} + |x - z|)
\end{aligned}$$

where the second equality follows the fact that $\text{dist}(x, [a, b]) = \min_{y\in[a,b]} |x - y|$ and the third equality from the definition of $\text{dist}(\cdot, \cdot)$. We now analyze two sub-cases separately.

Sub-case 1: $x \in [c, d]$ In this case, $\text{dist}(x, [a, b]) = \text{dist}(x, [b, c]) + (c - b)$ and we are done.

Sub-case 2: $x \notin [c, d]$

Then,

$$\min_{z\notin[c,d]} (\min\{|z - c|, |z - d|\} + |x - z|) = \text{dist}(x, [c, d]),$$

since the minimizer is found by setting $z = x$. From the above equality,

$$\begin{aligned}
&\min_{\substack{y\in[a,b]\\ z\notin[c,d]}} (\text{dist}(y, [a, b]) + \text{dist}(z, [c, d]) + |x - y| + |x - z|) \\
&= \text{dist}(x, [a, b]) + \text{dist}(x, [c, d]) \\
&\geq \text{dist}(x, [b, c]) + (c - b),
\end{aligned}$$

where the inequality again follows from (2).

Case 3: $y \notin [a, b]$ and $z \notin [c, d]$

In this case, by the definition of $\text{dist}(\cdot, \cdot)$

$$\begin{aligned}&\min_{\substack{y \notin [a,b] \\ z \notin [c,d]}} (\text{dist}(y, [a, b]) + \text{dist}(z, [c, d]) + |x - y| + |x - z|) \\ &= \min_{\substack{y \notin [a,b] \\ z \notin [c,d]}} (\min\{|y - a|, |y - b|\} + \min\{|z - c|, |z - d|\} \\ &\qquad\qquad + |x - y| + |x - z|).\end{aligned}$$

We now analyze two sub-cases separately.

Sub-case 1: $x \notin [a, b]$ and $x \notin [c, d]$

Now, by the same reasoning as before,

$$\begin{aligned}&\min_{y \notin [a,b]} (\min\{|y - a|, |y - b|\} + |x - y|) = \text{dist}(x, [a, b]) \\ \text{and } &\min_{z \notin [c,d]} (\min\{|z - c|, |z - d|\} + |x - z|) = \text{dist}(x, [c, d]),\end{aligned}$$

since the minimizer is found by setting $y = x$ and $z = x$. Thus, by independence of the terms y and z,

$$\begin{aligned}&\min_{\substack{y \notin [a,b] \\ z \notin [c,d]}} (\text{dist}(y, [a, b]) + \text{dist}(z, [c, d]) + |x - y| + |x - z|) \\ &= \text{dist}(x, [a, b]) + \text{dist}(x, [c, d]) \\ &\geq \text{dist}(x, [b, c]) + (c - b),\end{aligned}$$

where the second inequality follows from (2).

Sub-case 2: $x \in [a, b]$ OR $x \in [c, d]$

By symmetry, without loss of generality we assume that $x \in [a, b]$. Since the two intervals are disjoint $x \in [a, b]$ implies $x \notin [c, d]$. Then since $x \notin [c, d]$,

$$\min_{z \notin [c,d]} (\min\{|z - c|, |z - d|\} + |x - z|) = \text{dist}(x, [c, d])$$

since the minimizer is realized by setting $z = x$. Finally since $\text{dist}(x, [a, b]) = 0$,

$$\begin{aligned}&\min_{\substack{y \notin [a,b] \\ z \notin [c,d]}} (\text{dist}(y, [a, b]) + \text{dist}(z, [c, d]) + |x - y| + |x - z|) \\ &\geq \text{dist}(x, [c, d]) = \text{dist}(x, [a, b]) + \text{dist}(x, [c, d]) \\ &\geq \text{dist}(x, [b, c]) + (c - b),\end{aligned}$$

where the second inequality follows from (2). As this is the final case, we have proven the original claim. ∎

Lemma 2. *If $a \leq b$, $c \leq d$, $a \leq c$, and $b \geq c$ are integers, then for all integers x the following inequality holds:*

$$\min_{y,z \in \mathbb{Z}} (dist(y, [a, b]) + dist(z, [c, d]) + |x - y| + |x - z|) \geq dist(x, [c, b]).$$

Proof. The proof again follows by a case analysis, but it is much simpler than in (Lemma 1).

Case 1: $x \in [c, b]$

This case is trivial since $\text{dist}(x, [c, b]) = 0$ and the left hand side of the inequality is always non-negative.

Case 2: $x \leq c$ or $x \geq b$

As these cases are symmetric, it suffices to only consider the former case where $x \leq c$. First, if $z \leq c$,

$$\begin{aligned} \text{dist}(z, [c, d]) + |x - z| &\geq |x - c| \\ &\geq \text{dist}(x, [c, b]) \end{aligned}$$

since the minimizer is found when $z \in [x, c]$. Second, if $z \geq c$,

$$|x - z| = z - x \geq |c - x| \geq \text{dist}(x, [c, b]),$$

since z is to the right of c while x is to the left of c. This completes the proof. ∎
