# Supplementary figures and images for: A zero-agnostic model for copy number evolution in cancer
Source: PLoS Comput Biol. 2023 Nov 9;19(11):e1011590. doi: 10.1371/journal.pcbi.1011590 (PMC10662746; doi:10.1371/journal.pcbi.1011590)

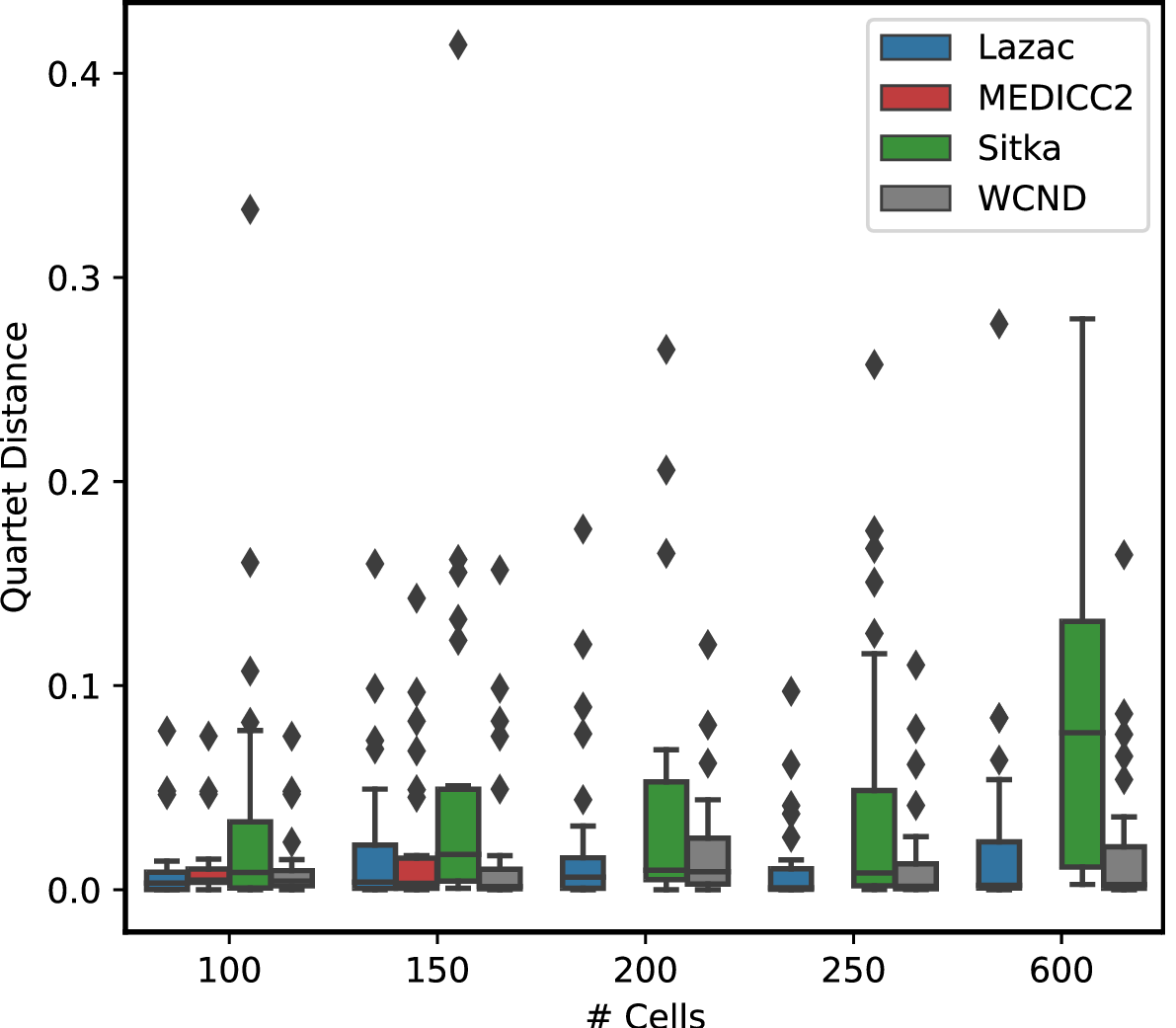

Supplement: S1 Fig — The exact versus the relaxed score of the optimal solution to the ZCNT small parsimony problem when the balancing condition is removed across 200 phylogenies. The 200 phylogenies were obtained by stochastic perturbation of the phylogeny inferred Sitka [39] on sample SA1053. The dotted line is computed by performing linear regression and is defined by y = 0.9313 * x + 204.1 with an R2 = 0.972 and a p = 1.05 * 10−156. (TIFF) [file pcbi.1011590.s006.tiff]

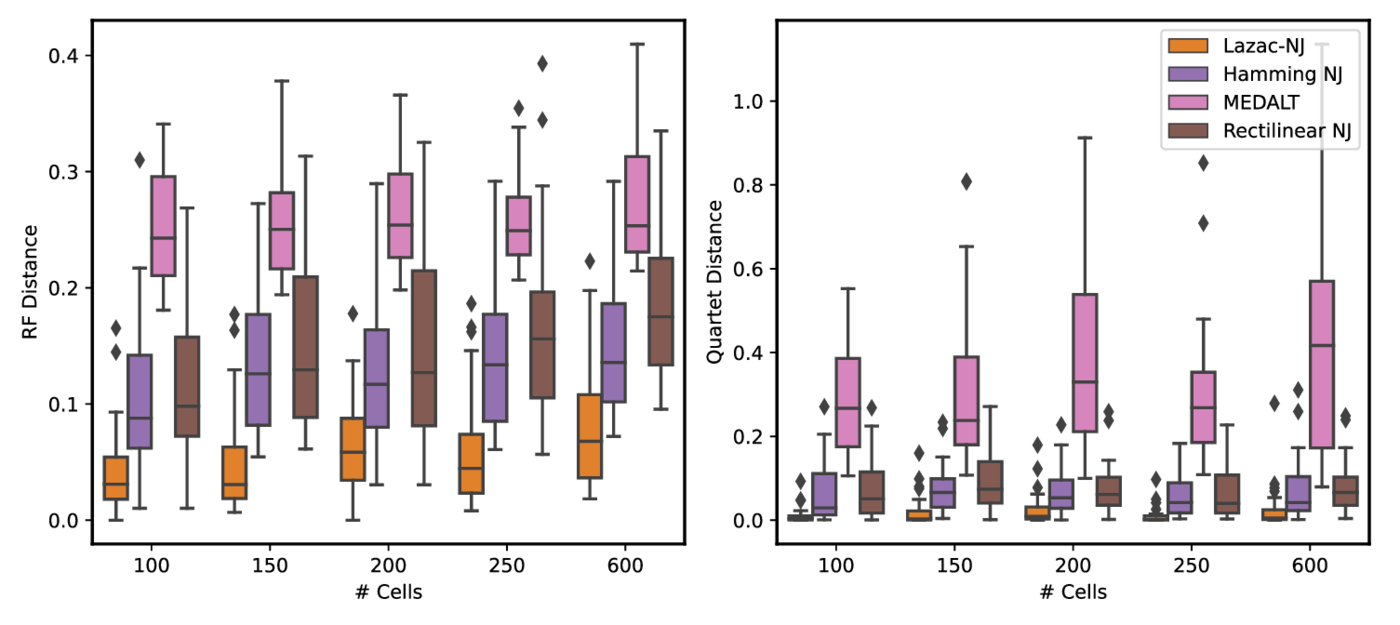

Supplement: S2 Fig — Baseline reconstruction accuracy on (left: RF distance; right: Quartet distance) CONET simulated data across simple NJ methods for copy number tree reconstruction with varying number of cells n = 100, 150, 200, 250, 600 across four sets of loci l = 1000, 2000, 3000, 4000 and seven random seeds s = 0, 1, 2, 3, 4, 5, 6. (TIFF) [file pcbi.1011590.s007.tiff]

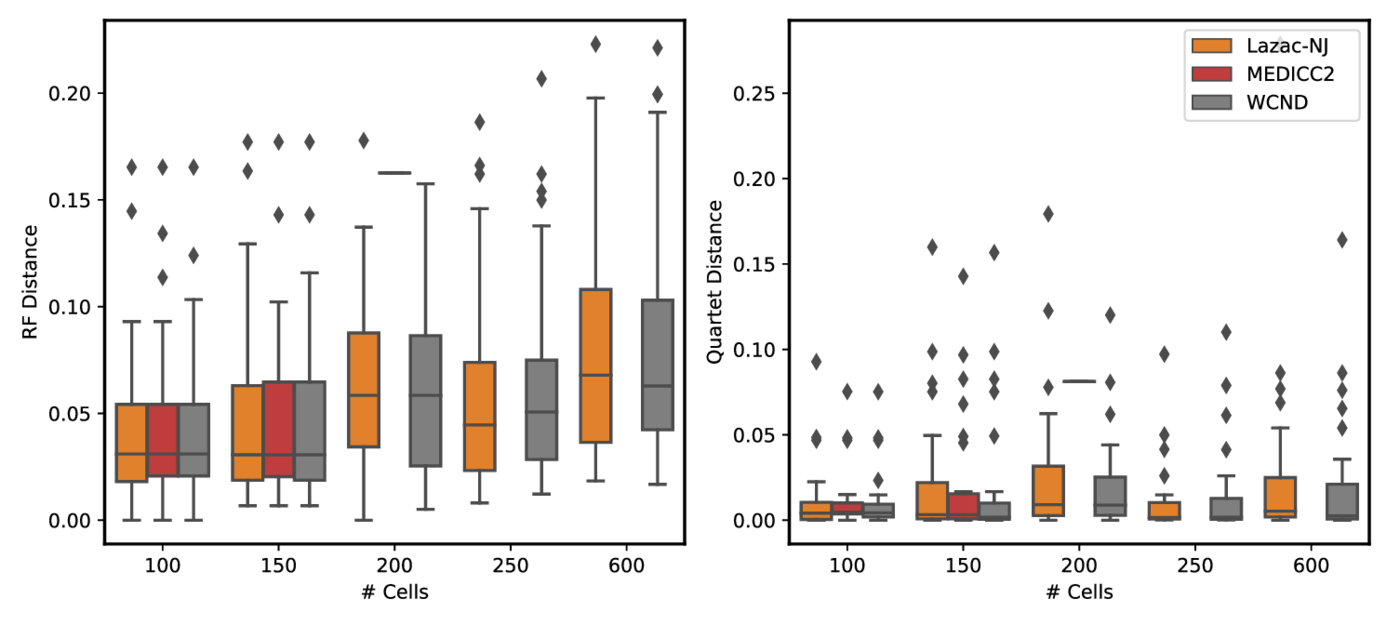

Supplement: S3 Fig — Comparison of reconstruction accuracy (left: RF distance; right: Quartet distance) CONET simulated data across distance based methods for copy number tree reconstruction with varying number of cells n = 100, 150, 200, 250, 600 across four sets of loci l = 1000, 2000, 3000, 4000 and seven random seeds s = 0, 1, 2, 3, 4, 5, 6. As MEDICC2 was too slow to run on more than 150 cells, we exclude it from comparisons where the number of cells n > 150. (TIFF) [file pcbi.1011590.s008.tiff]

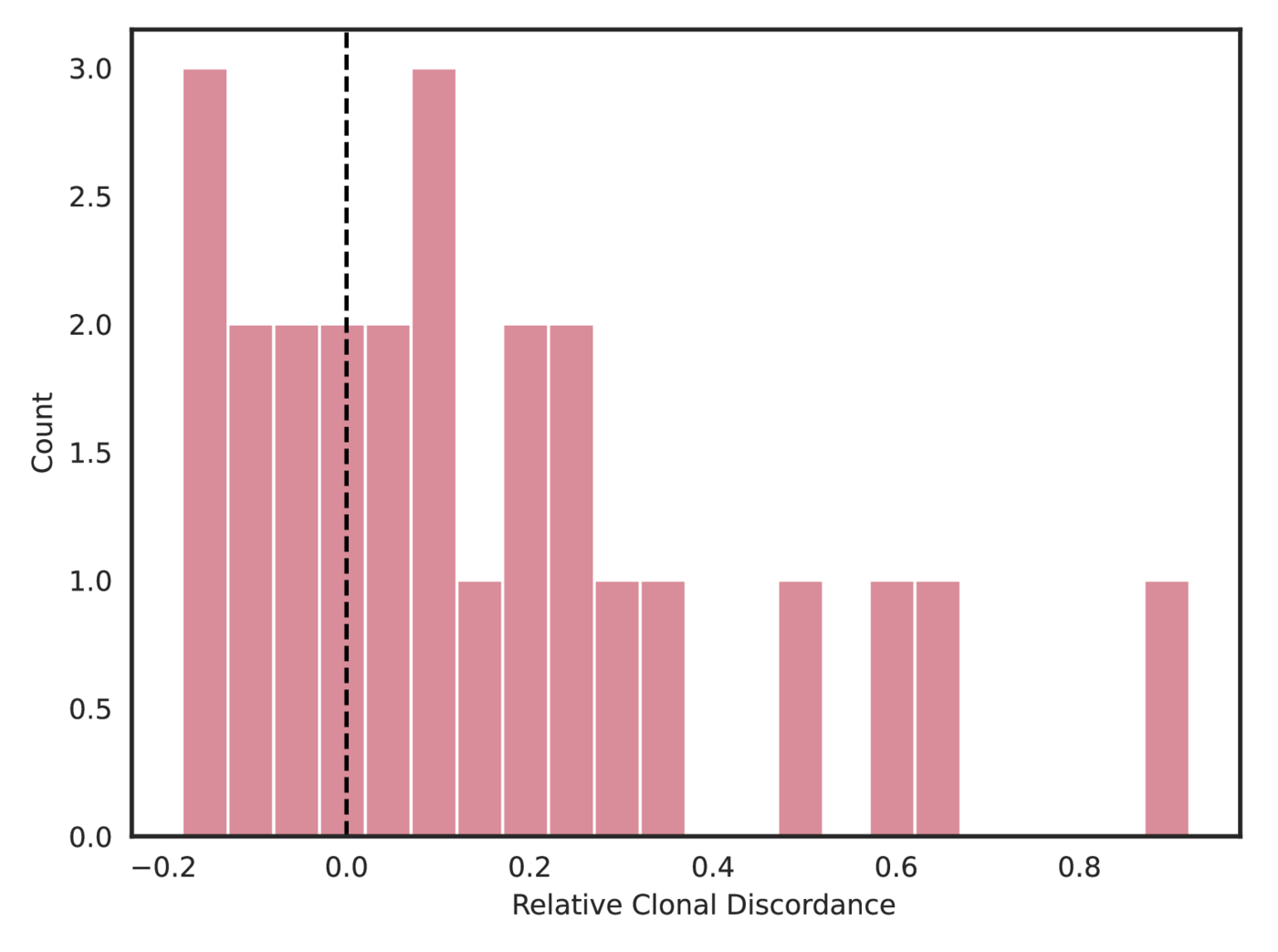

Supplement: S4 Fig — The relative clonal discordance score p2-p1p1+p2 where p1, p2 are the clonal discordance scores of the Lazac and Sitka inferred phylogenies respectively. (TIFF) [file pcbi.1011590.s009.tiff]

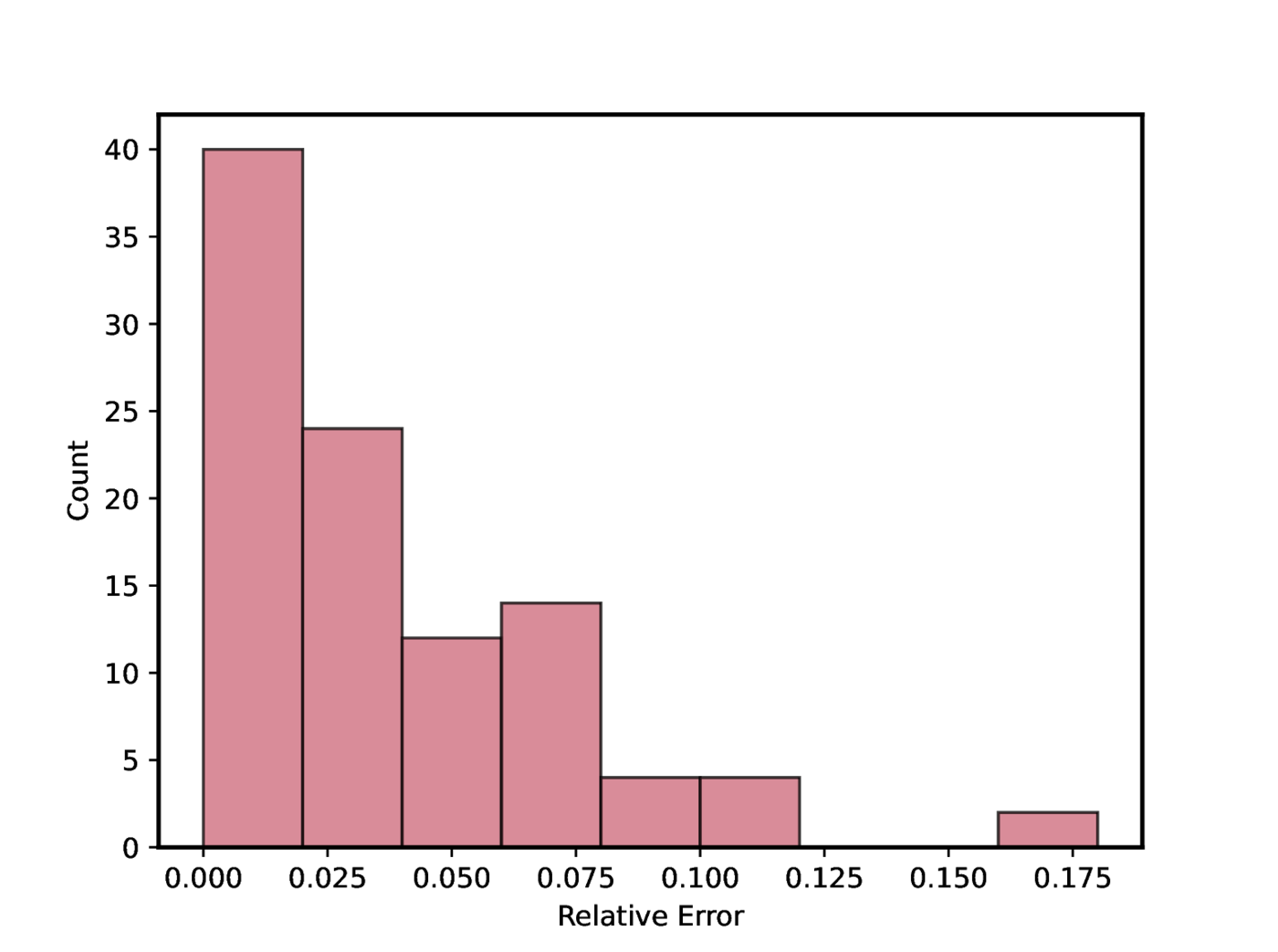

Supplement: S5 Fig — Relative difference between the ZCNT distance d(p, p′) and the CNT distance computed for patient 8 from a metastatic prostate cancer tumor sample [38]. (TIFF) [file pcbi.1011590.s010.tiff]

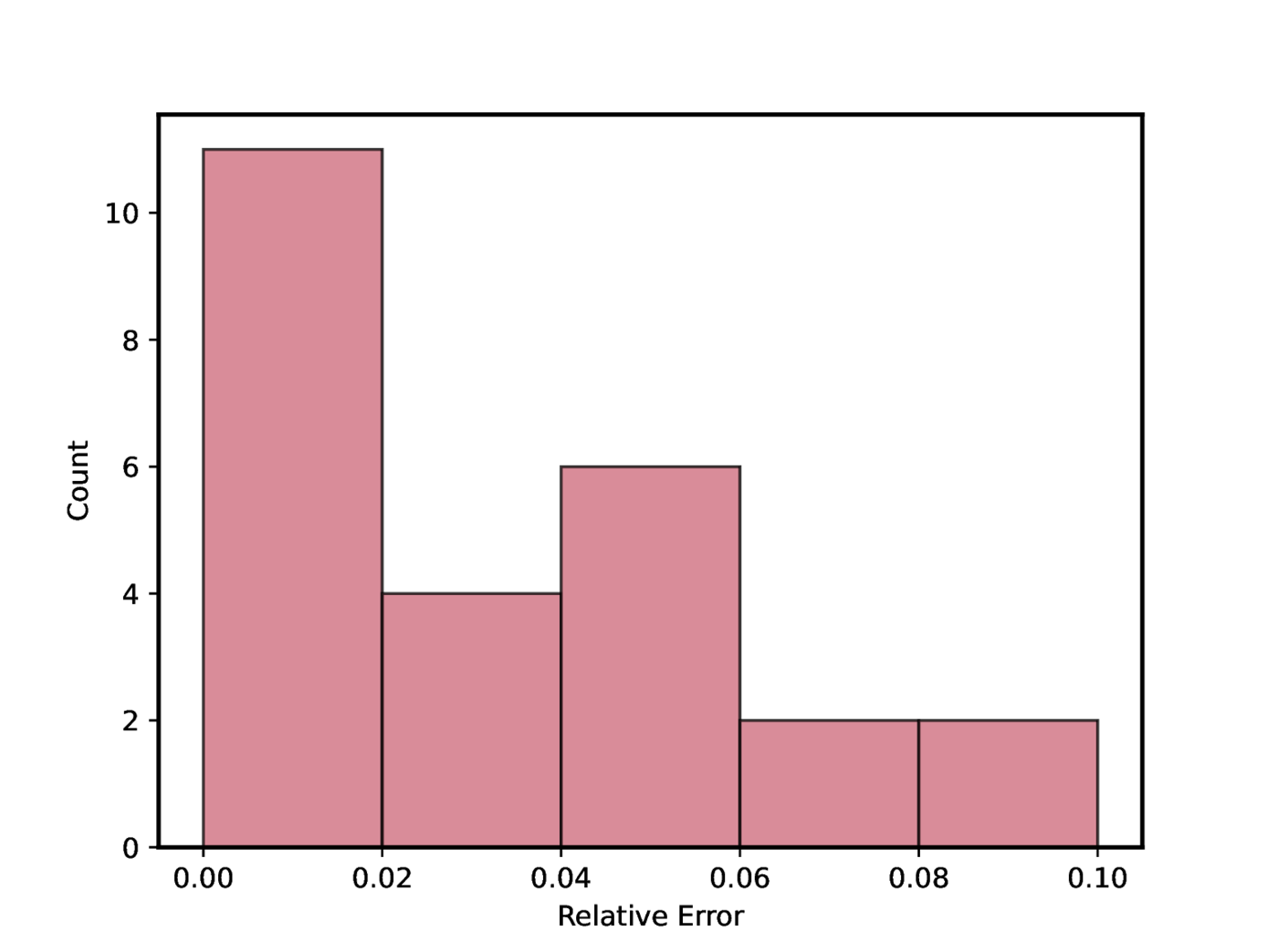

Supplement: S6 Fig — Relative difference between the ZCNT distance d(p, p′) and the CNT distance for patient 12 from a metastatic prostate cancer tumor sample [38]. (TIFF) [file pcbi.1011590.s011.tiff]

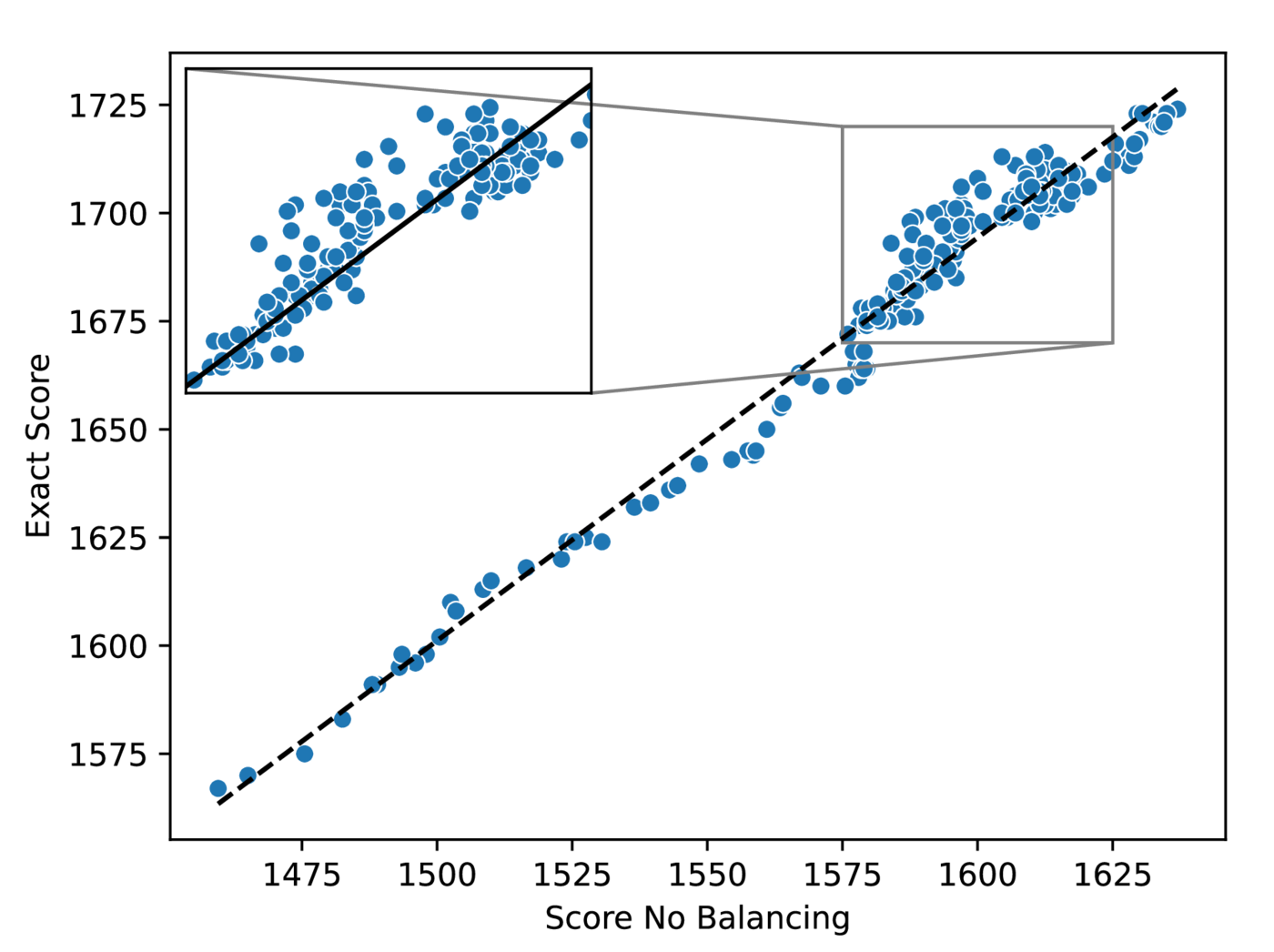

Supplement: S7 Fig — The exact versus the relaxed score of the optimal solution to the ZCNT small parsimony problem when the balancing condition is removed across 200 phylogenies. The 200 phylogenies were obtained by stochastic perturbation of the phylogeny inferred Sitka [39] on sample SA1053. The dotted line is computed by performing linear regression and is defined by y = 0.9313 * x + 204.1 with an R2 = 0.972 and a p = 1.05 * 10−156. (TIFF) [file pcbi.1011590.s012.tiff]

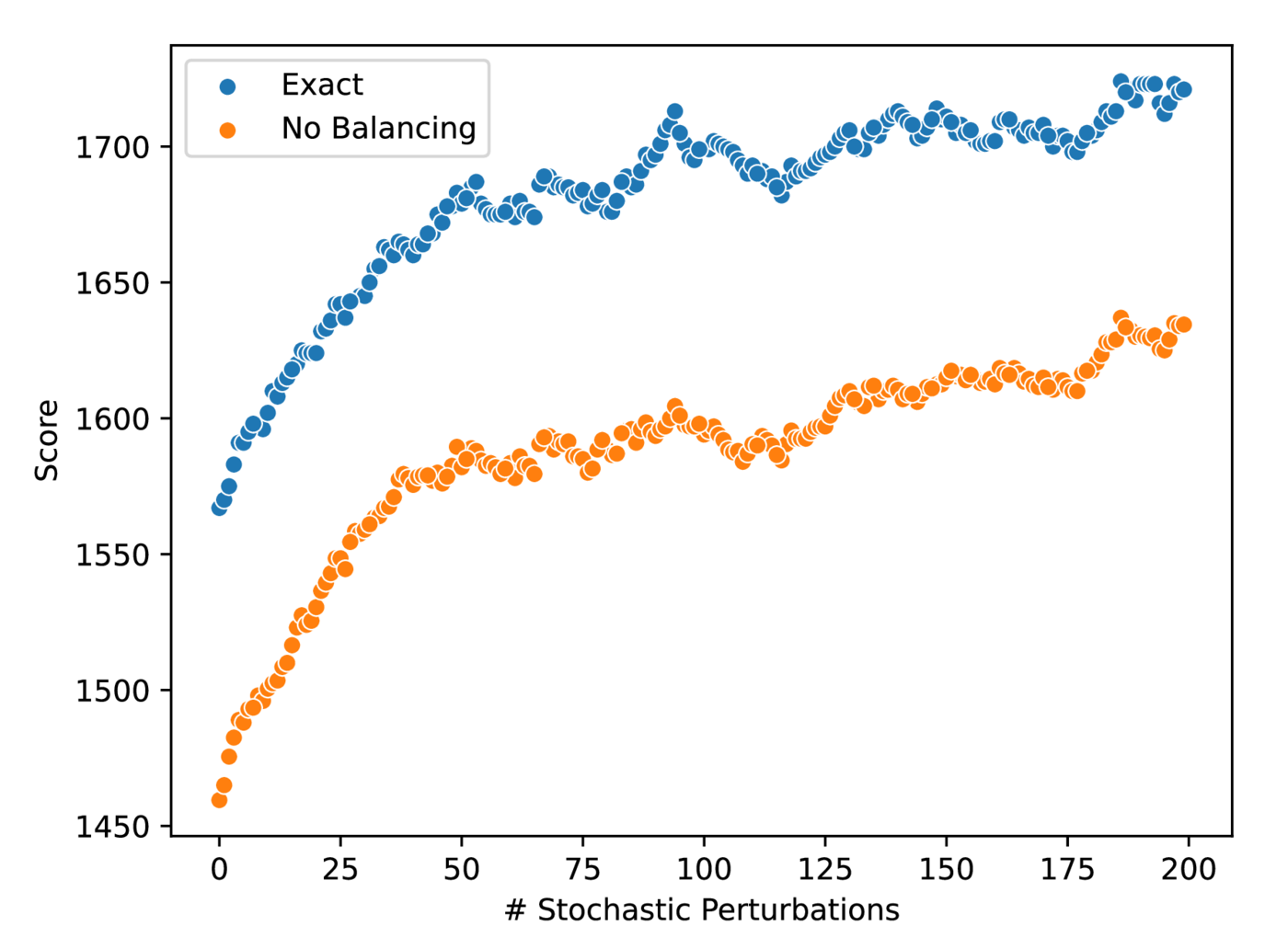

Supplement: S8 Fig — The exact and relaxed scores of the optimal solution to the ZCNT small parsimony problem as a function of the number of stochastic perturbations applied to the phylogeny inferred by Sitka [39] on sample SA1053. (TIFF) [file pcbi.1011590.s013.tiff]

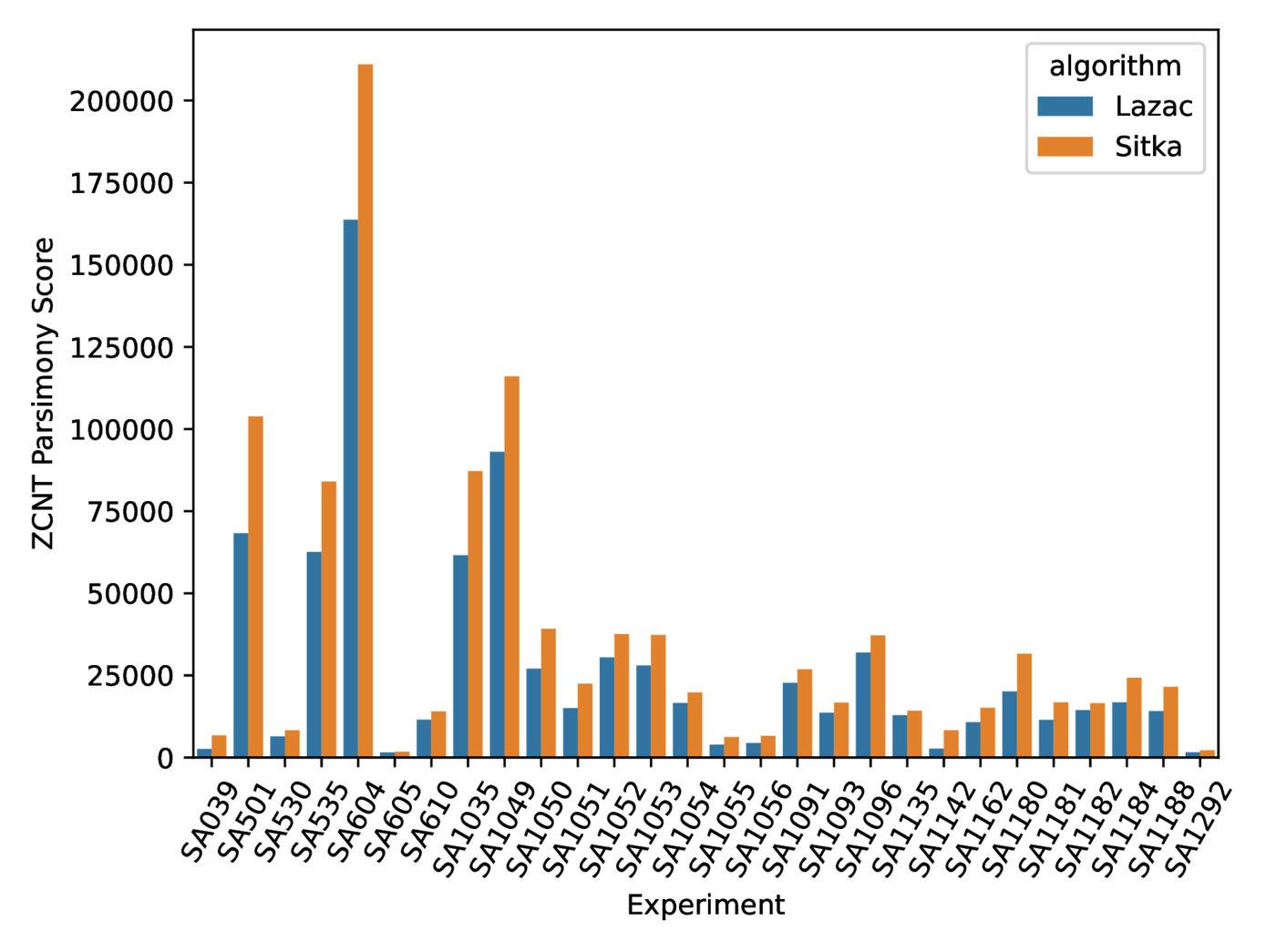

Supplement: S9 Fig — The ZCNT parsimony score for the optimal solution to the ZCNT small parsimony problem for Lazac and Sitka inferred phylogenies on copy number profiles from 28 human breast and ovarian tumour samples [7]. (TIFF) [file pcbi.1011590.s014.tiff]

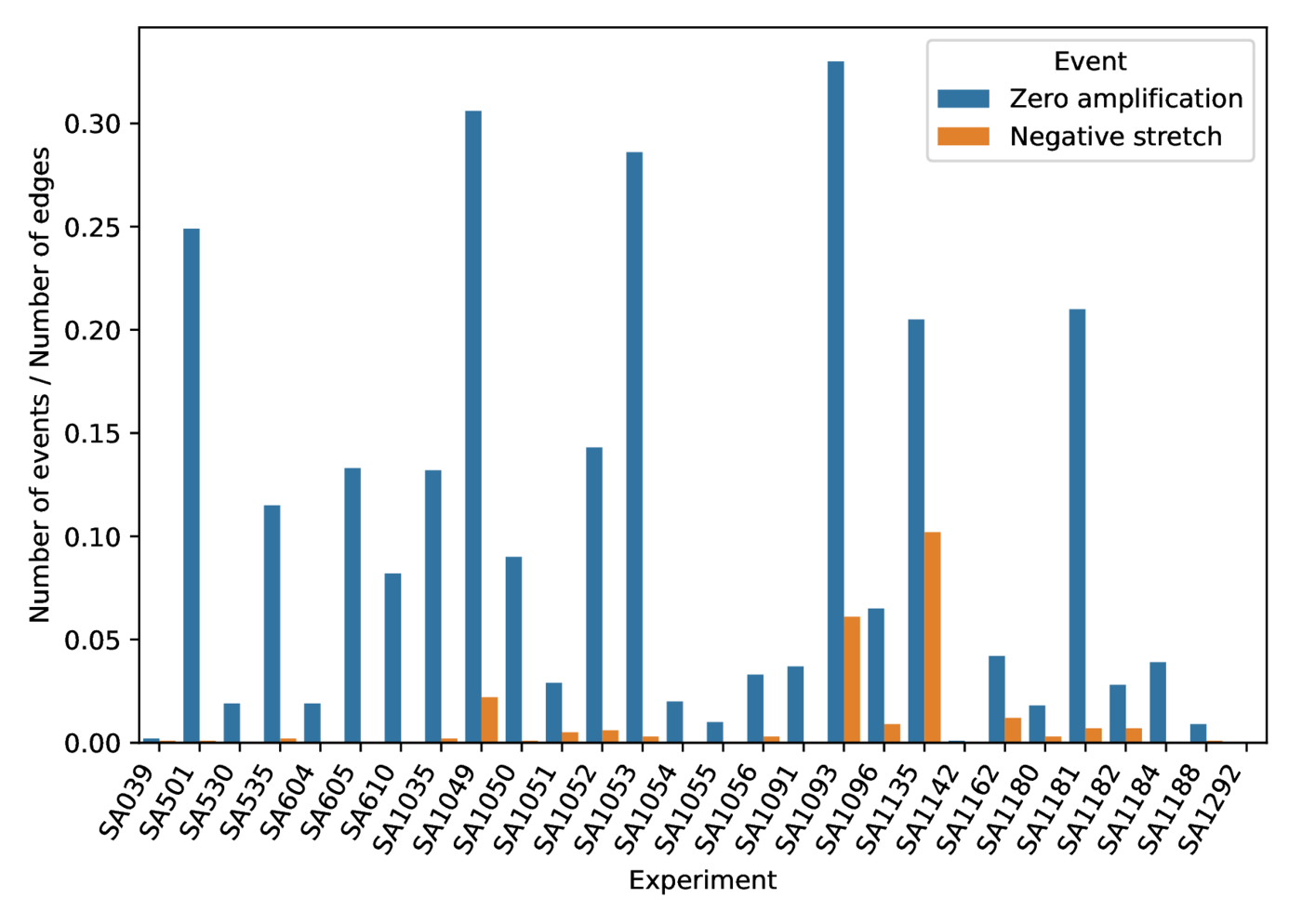

Supplement: S10 Fig — The edge normalized count of zero amplification and negative stretch events in the ancestral labelings of Lazac inferred phylogenies on copy number profiles from 28 human breast and ovarian tumour samples [7]. (TIFF) [file pcbi.1011590.s015.tiff]

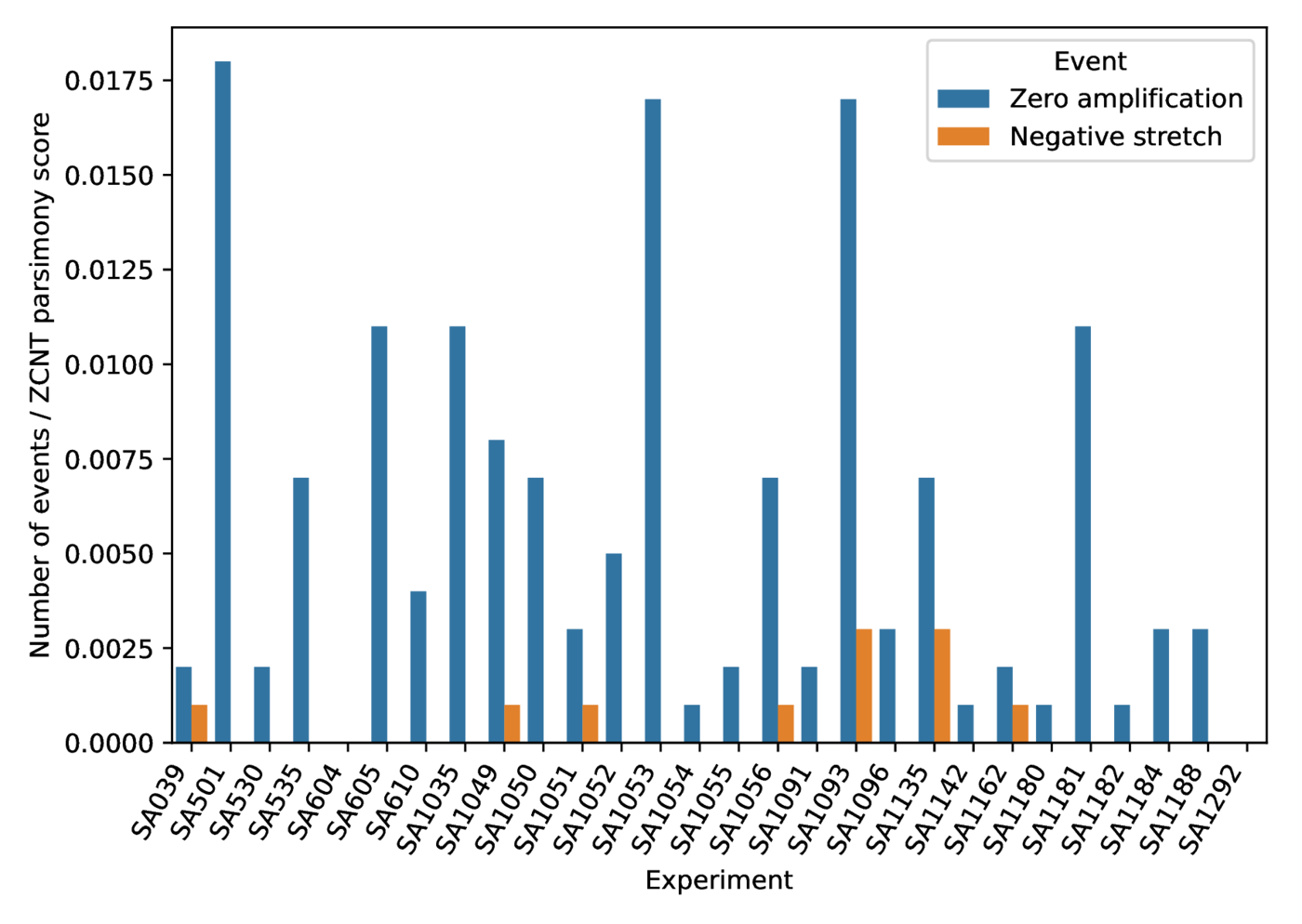

Supplement: S11 Fig — The ZCNT parsimony score normalized count of zero amplification and negative stretch events in the ancestral labelings of Lazac inferred phylogenies on copy number profiles from 28 human breast and ovarian tumour samples [7]. (TIFF) [file pcbi.1011590.s016.tiff]

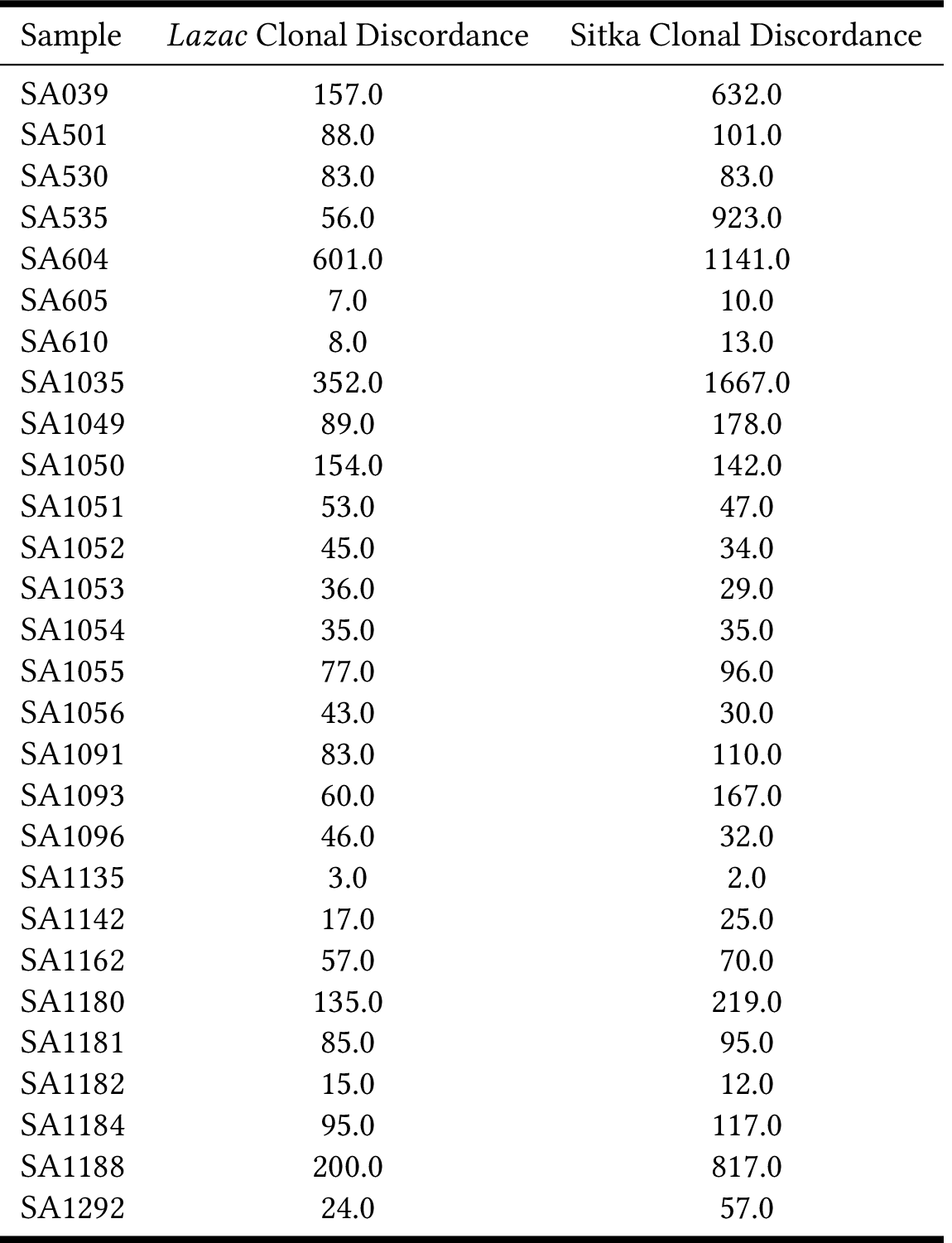

Supplement: S12 Fig — Comparison of Lazac and Sitka clonal discordance scores across 28 human breast and ovarian tumour samples [7]. (TIFF) [file pcbi.1011590.s017.tiff]

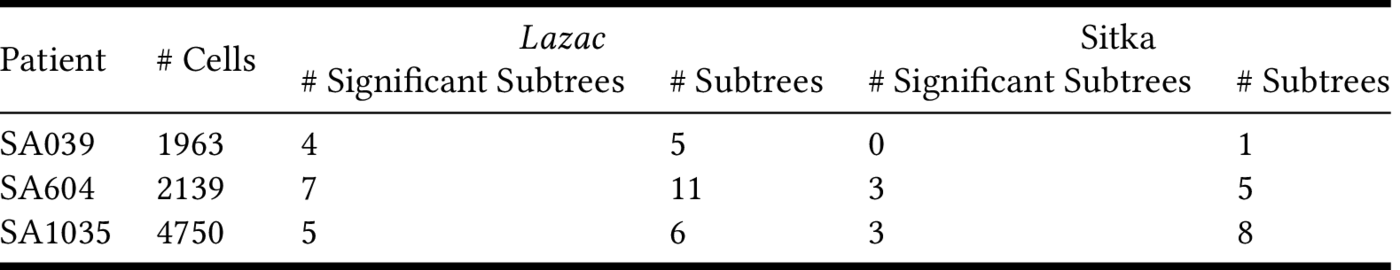

Supplement: S13 Fig — Results of somatic SNV analysis on subset of human breast and ovarian tumour samples [7]. Subtrees were called significant if the SNV permutation test p-value was below 0.05 for that clone. (TIFF) [file pcbi.1011590.s018.tiff]
