# Supplementary material for: A zero-agnostic model for copy number evolution in cancer
Source: PLoS Comput Biol. 2023 Nov 9;19(11):e1011590. doi: 10.1371/journal.pcbi.1011590 (PMC10662746; doi:10.1371/journal.pcbi.1011590)
Supplement: Attachment — Submitted filename: Reviewer Response.pdf [file pcbi.1011590.s019.pdf]

We thank the reviewers for their positive feedback and insightful comments. Since the submission of the manuscript in January, we have made significant revisions which we believe substantially improve the quality of the paper:

1. We have renamed the Resurrecting Copy Number Transformation (RCNT) Model to the Zero-agnostic Copy Number Transformation (ZCNT) model, as we believe it better matches the key assumptions of the work which is that zero copy number loci are not treated differently. This has resulted in a change in title.
2. We have cleaned up and streamlined the theory of ZCNT to improve the clarity of the manuscript.
3. Per Reviewer 2's comments, we performed an in-depth analysis of the "artifacts" that result from relaxing the ZCNT small parsimony problem by comparing the relaxed and exact solutions of the ZCNT small parsimony problem on real data. This is included in the new Section 7.4.
4. We have extended the analysis of single-cell copy number phylogenies from the data in (Funnel et al. 2022) present in the paper by including new pieces of quantitative analysis and an in-depth qualitative analysis of patient sample SA1292.
5. We have extended the theory of ZCNT:
    a. We have derived a new 2-approximation algorithm for ZCNT small parsimony.
    b. We have a new polynomial time dynamic programming algorithm for the special case of ZCNT small parsimony when the tree takes a star shape (also known as the median problem).

As such, we believe the new manuscript adequately responds to the reviewer comments. Please note that some of these changes were made following the RECOMB-CCB reviews in a version that we posted to bioRxiv on April 12, 2023. The changes from the bioRxiv version of the paper are marked in red in the manuscript file.

The zero-agnostic copy number transformation model

Reviewer Response

---------------------- REVIEW 1 --------------------

The paper presents a relaxation over the widespread Copy Number Transformation (CNT) model, the Resurrecting Copy Number Transformation (RCNT). This simplified model allows for a polynomial solution to the small parsimony problem (upon relaxation of some constraints).

I find the paper to be well written, with a consistent logical flow starting from the base concepts and functions used in the CNT, and ending with the definitions of the RCTN, delta profiles, and the distance calculation.

As a suggestion to the authors, I would probably make a more exhaustive figure 1, with an example of an actual tree, that maybe shows some example of the calculation and of the property presented in the paper.

We agree with the reviewer and have updated (Figure 1) to be more comprehensive, showing both an example of the model and the ZCNT small parsimony problem.

I believe the RCNT, where the main difference with the CNT is the possibility of a copy number transformation to alter a segment, to be a sensible approximation to the real model and I also expect 0 copy number region to be relatively rare and marginal to the final tree inference (at least for most of the cases).

Please note that in response to comments of Reviewer 2 below, we further investigated the biological plausibility of our model on real patient samples from (Funnell et al. 2022) and found that ZCNT events violating the CNT model were rare.

The algorithm has good performances in terms of reconstruction accuracy when compared to other state-of-the-art tools and manages to achieve a consistent speed-up (100x). Given the speed at which the scDNA-seq field is evolving, I foresee a wide adoption of fast and accurate algorithms for CNV tree reconstruction.

This piece of work also offers interesting starting points for further research, both in the better algorithmic characterization of the RCNT and/or other similar relaxed versions of CNT.

Considering all of this, I support the paper for acceptance.

---------------------- REVIEW 2 ---------------------

This paper develops a theoretical model and some associated algorithms for problems in evolutionary distance information from copy number data and its use in tumor phylogenetics. It is a theoretically interesting paper, in proposing some relaxations of a popular but algorithmically problematic model for clonal evolution that lead to improved practical utility. It offers a creative solution to the problems of the copy number transformation (CNT) model, specifically in allowing for some seemingly biologically implausible but algorithmically convenient generalizations of the model. These are then applied to find better solutions to the small parsimony problem and, through a simple search heuristic, to the large parsimony problem. The work is well motivated in trying to find more efficient ways of handling the computational intractability of some core phylogeny problems on copy number variation, essentially by making the measure apparently less realistic to yield a metric and allow for independence across variants. There is a very nice empirical analysis on both real and simulated data, providing good evidence that the method is effective in practice and improves both accuracy and runtime relative to the standard CNT model. The work appears likely to have practical value in analyzing copy number data and may inspire future theoretical investigations. There is a lengthy appendix, which I think adds useful

details on the methods and additional results, but the main paper is readable without it. There are a few points on which I believe it could use clarification or correction, though:

1. I believe the paper could use more comment on what kinds of artifacts one might produce through the relaxations proposed, how problematic they are biologically, and whether one actually sees them frequently when applying the method. It seems pretty clear what "error" one is enabling by allowing transitions from zero to one copy of a gene, and perhaps it is an acceptable error that might legitimately be observed due to missing data. I am less clear on what artifacts one might see if one drops the balancing condition and whether they would be particularly problematic if they did occur.

We thank the reviewer for this comment as it leads to important and interesting questions about the ZCNT model. As such, we first investigated the artifacts produced by the proposed relaxations on single-cell whole genome sequencing data (Funnel et al. 2022) and reported our findings in a new section of the paper (Section 7.4). Briefly, we found that in practice, relaxing the integrality condition has no effect on the ZCNT small parsimony score (that is, solutions are always integral in practice), while relaxing the balancing condition does alter the score. However, the ranking of phylogenies with and without the the balancing condition are highly concordant (Spearman R^2 = *0.9644)* meaning that relaxation of the balancing condition is suitable for phylogenetic inference. These findings are summarized in the revised manuscript as follows.

"We investigated the approximation error produced by the relaxations (Section 5) used in our two polynomial time algorithms for the ZCNT small parsimony problem. To perform this investigation, we first generated a set of 200 copy number phylogenies by perturbing the published phylogeny [34] using from 1 to 200 random nearest neighbor interchange (NNI) operations (Section 7.3). Then, for each phylogeny, we computed the optimal solution to the ZCNT small parsimony problem and its two relaxations using (integer) linear programming.

Importantly, we found that the exact solution to the ZCNT small parsimony problem and the solution obtained by relaxing the integrality condition were identical in every case. This leads us to believe that the relaxed linear program often has a special structure, which makes it a good approximation to the ZCNT small parsimony problem.

Removing the balancing condition resulted in solutions with a lower score, implying that the balancing condition does meaningfully constrain the solution space. However, the rankings of phylogenies with and without the balancing condition were highly concordant (Spearman correlation $R^2 = 0.9644$, $p < 10^{-100}$) across the 200 copy number phylogenies (Supplementary Figure 7, 8). Thus, when ranking phylogenies by ZCNT parsimony score – as is done when solving the ZCNT large parsimony problem – removing the balancing condition does not change the result substantially."

To the question of the biological plausibility of the ZCNT model as compared to the CNT model, we checked whether the ZCNT model attributed negative copy numbers to ancestors or

included amplifications of regions with zero copy number. In brief, we found that for all 28 patient samples we analyzed: i) non-negativity violations were infrequent in most samples; (ii) ZCNT events violating the CNT model made up less than 2% of the total ZCNT events in the most parsimonious ancestral labeling. Thus, it is reasonable to use the ZCNT as opposed to the CNT model for ranking candidate trees. This analysis is described in paragraph 6 of (Section 7.3):

"Fourth, we verified that the ancestral labeling inferred by our ZCNT model was biologically meaningful by checking whether it assigned negative copy numbers to ancestors or included amplifications of regions with zero copy number. Negative copy numbers and amplification of zero copy numbers are allowed by the ZCNT model but are violations of the CNT model. In 10/28 patient samples, none of the ancestors were labeled with negative copy number regions and in all 28 samples, none of the ancestors were labeled with a copy number below -1. Copy number of -1 was extremely infrequent: across all samples, the total number of negative stretches normalized by the number of edges in the tree was at most 0.1 (median 0.002) (Supplementary Figure 10). Amplifications of zero (or negative) copy number loci were also quite rare: across all samples, the total number of such amplifications normalized by the number of edges in the tree was less than 0.33 (median 0.042) (Supplementary Figure 10). These CNT violations had negligible effect on the parsimony score: for all samples, CNT violations contributed less than 2% of the ZCNT small parsimony score (Supplementary Figure 11). Thus, while our ZCNT model allows for both amplifications of zero copy number loci and negative copy number loci, these events were rare on the patient samples we analyzed and contributed little to the ZCNT small parsimony score."

2. In a related point, I was unclear on whether the clonal discordance score was allowing for the "impossible" events enabled by the relaxations. For example, is an increase from zero to one copy of an interval counted as one clonal transition in computing the clonal discordance score? I was not sure one way or another, but it could be an unfair test if it is implemented in such as way as to treat the unrealistic transitions enabled by the relaxations as single moves for the parsimony measure.

This is a reasonable question. The clonal discordance score is not directly related to the models of copy number evolution (CNT and ZCNT). Instead the clonal discordance is a measure of agreement between the inferred trees and the copy number clones inferred in the original publication of this single-cell data (Funnell et al. 2022). Specifically, the clonal discordance score for a tree T is the minimum number of changes in clone label that occur on T when the leaves are labeled with the published clone labels. The clone label is a single phylogenetic character with a median of 8 states across all samples, and is the result of clustering the copy number profiles of the cells.

Since it was not clear how the clonal labeling was defined, we added the following sentence to the paper, which hopefully clears up the confusion:

“The clone labels are obtained from the assignment of each cell to one of k clones, defined by the clustering of cells according to their copy number profiles performed in (Funnell et al. 2022).”

3. The large parsimony method relies on a very simple local search heuristic using the nearest-neighbor interchange (NNI) Monte Carlo move and it seems likely one could do a lot better than this. This method is not really the main point of the paper so I think it is fine to have something simple as a demonstration. I think it is worth commenting on whether more state-of-the-art ideas in phylogenetics might also be adapted to use RCNT distance developed here.

We agree that there are many other tree search heuristics in the literature and that different heuristics might lead to better results. However, we note that our local search procedure is nearly identical to IQ-TREE 2’s search procedure (Minh et al. 2020), which is a recent and highly cited method for phylogenetic tree inference, and is described in another publication on lineage tracing (Sashittal et al. 2023; doi:10.1101/2022.12.18.520935).

To address the reviewer’s comment we added the following mention of future work to the Discussion.

“Second, the algorithm we developed for the ZCNT large parsimony problem relies on a simple, hill climbing search using nearest-neighbor interchange operations. We expect that a more advanced approach that uses state-of-the-art techniques from phylogenetics [27, 31] could substantially improve both inference speed and accuracy.”

4. The authors mention that the RCNT model is not biologically realistic, but performs well compared with the regular CNT model, which is more biologically realistic. It might be worth discussing some alternative distance metrics for both the copy-number transformation model and alternative evolutionary models. There may also be some value in discussing the biological plausibility of various common evolutionary distance metrics and how such metrics can impact various phylogenetic reconstructions in the introduction section.

We thank the reviewer for the suggestion and have described other distance metrics used to compute copy number phylogenies in the introduction to (Section 2).

“One of the most basic phylogenetic principles is that nearly perfect measurement of evolutionary distances enables exact recovery of the evolutionary history [39] . It is thus not surprising that many of the successful attempts at inferring copy number phylogenies focus on finding good methods to compute an evolutionary distance between a pair of copy number profiles. Early methods for computing evolutionary distances on copy number data [3, 7] employed simple measures of distance such as the Hamming, weighted Hamming, and ℓ_1 distance between copy number profiles. However, these distances do not account for dependencies between loci caused by long CNAs spanning contiguous segments of the genome, leading to inaccurate phylogenetic reconstruction [37, 22].”

I also noted one minor error: in the Figure 1 caption, there is a placeholder to be filled in "(denoted by ????)".

Figure 1 and its caption has been completely updated per Reviewer 1's suggestion, which also addresses this issue.

---------------------- REVIEW 3 --------------------
Resurrecting the copy number transformation model (RCNT) is a generalization of the existing CNT model. While both models define a distance between copy number profiles and are used for the reconstruction of copy number phylogenies, the RCNT model allows for events that "resurrect" deleted genomic regions. This biologically unrealistic simplification of the model enables faster inference of good quality copy number phylogenies, demonstrated both on real and simulated data. This is achieved by developing algorithms for solving (relaxed) small and large parsimony problems.

General:

1. Nomenclature for the "I" index – it denotes different across the text: sometimes it's bins, sometimes loci, and less directly - also segments or genes. I suggest unifying this.

We updated the text to use the unified language "loci."

2. Figure 2 (and similarly Sup Figures 1, 2, 3) – the footnote states that the results are shown for varying no of cells and loci – but all the plots only show different no of cell setups (on the X axes). I assume that the simulations were done for varying no of loci and the results were averaged per the same no of cell setups. But then none of the figures illustrates the sensitivity to the no of loci as the footnote is suggesting. Also, only the footnote for SF3 explains why MEDICC2 results only appear for 100 and 150 cells.

We modified the figure's caption to reflect that the simulations were done across four different settings of the number of loci and seven random seeds, but only plotted across against the number of cells. Said another way, each boxplot contains the result of 28 simulations across 7 random seeds and 4 different settings of the number of loci. We also added a sentence to the main text to explain that MEDICC2 did not scale beyond 150 cells.

3. For results comparison, authors use RF and Quartet distances. First, none of those two measures is defined, second – there should be a short explanation of the difference in those two distances and an explanation of why sometimes both are shown (for example Supp F2 and F3), and in other cases only one is presented (Figure 2 – RF distance, Supp F1 – Quartet distance).

We added a section in the supplement (Supplementary Methods A.5) that defines both distances. We also added references to this section, where relevant, in the main text. The reason that both are not shown in (Figure 2) is due to space constraints. However, we placed the corresponding Quartet distance figure in the supplement (Supplementary Figure 1) as a standalone figure and referenced it in the main text along with (Figure 2).

4. Section 6.3, last paragraph: I suggest adding per-subtree coverage info – this would be more informative than the percentage of cells in the context of SNV inference.

Thank you, we have added per-subtree coverage info as suggested.

Editorial:

1. Page 2, is: „can alter the number of copies a large section of the genome", should be: „can alter the number of copies of a large section of the genome".

Fixed.

2. Page 2, paragraph 4, in the sentence where the complexity of the algorithm is introduced, there is an explanation of what "B" denotes, but there also should be an explanation for "n", which was not introduced before. "n" is introduced at the beginning of Section 2, but indeed explanation of B is not mentioned there, but it is explained for the second time at the end of Section 2.

Fixed.

3. Sitka [35] – Where was it published or is it a preprint? – needs more clarification in Bibliography.

This is a bioRxiv preprint. We have added clarification in the bibliography.

4. Page 3, at the beginning of Section 2 the first "p" is missing the "j" subscript.

The first "p" at the beginning of Section 2 is a vector and does not need a subscript.

5. Page 2, last paragraph, is: "a segment-based (i.e. non-independence) model of copy number evolution", should be: "a segment-based (i.e. non-independent) model of copy number evolution" if this non-independence describes the model. If it describes sth else – it should be stated.

Fixed.

6. Page 3, end of page " (i.e. pi =0)" should have a comma at the end.

Fixed.

7. Page 4, in Problem 1, is: "[52, 44] shows there is…", should be "[52, 44] show there is…",

Fixed.

8. Page 4, next sentence starts with “Unfortunately however”, I suggest one of those (either unfortunately or however) is sufficient.

Fixed.

9. Page 4, at the beginning of Section 3 there is a sentence: “that describes the evolution of the tumor.’ I don’t think „tumor” is necessary here, since the same would be valid for any other group of related cells/organisms/entities.

You are correct, we have removed this clause to make the definition more general.

10. Page 4, Section 3, in the definition of cost J, no explanation of E(T) (in the sum index). E(T) is also used on page 8, problem 4 and throughout the Supplementary materials, but I could not find a definition for it. V(T) is also introduced and not defined, L(T) similarly. Mostly, the meaning of it can be guessed from the context, but I advise explicitly stating it.

Fixed.

11. Page 5, last paragraph of Section 3, there is an unnecessary “and” in “They also and formulated…”.

Fixed.

12. Page 7, missing comma in the sentence: „Clearly, if the sum is zero, the claim holds.”

Fixed.

13. Page 7, Corollary 2, is: „Given two copy number profiles u and v the copy number m profile minimizing”, should be: „Given two copy number profiles u and v, the copy number profile m minimizing”

Fixed.

14. Page 7, sentence: “Second, the number of copy number profiles are a priori unbounded,” is not the best put. Would reformulate to sth like: „The space of possible copy number profiles is a priori unbounded”.

Thank you for the suggestion to reformulate this sentence. We have taken your suggestion.

15. Page 8, second line, a missing comma before which.

Fixed.

16. Page 8, missing commas before: “the dynamic programming table has size…” and „we can solve this recurrence in…”.

Fixed.

17. Page 10, Figure 3 footnote – the sample is referred to as SA1184, but this ID is not mentioned in the main text while referring to this Figure. Either the footnote or/and the main text reference to the Figure should be updated such that it is clear which sample is illustrated. ALSO: Page 11, “We found that the phylogenies inferred by Breaked are substantially different than the phylogenies constructed by Sitka (Figure 3).” – Figure 3 only shows one phylogeny. I would add one sentence in the main text stating which of the 25 samples was used in Figure 3.

Fixed.
