# Supplementary material for: A zero-agnostic model for copy number evolution in cancer
Source: PLoS Comput Biol. 2023 Nov 9;19(11):e1011590. doi: 10.1371/journal.pcbi.1011590 (PMC10662746; doi:10.1371/journal.pcbi.1011590)
Supplement: Attachment — Submitted filename: PLOS-CB_ Reviewer Response Letter.pdf [file pcbi.1011590.s020.pdf]

PRINCETON UNIVERSITY

Ben Raphael
Professor of Computer Science
Princeton University
braphael@princeton.edu
609-258-6314

September 28, 2023

Dear Editor:

Please find the enclosed revision of our manuscript entitled "*A zero-agnostic model for copy number evolution in cancer,*" for consideration for publication in *PLoS Computational Biology*.

We were pleased to see enthusiasm for this work on part of the reviewers. We have revised the manuscript in response to the reviewer comments and provided a point-by-point response in attachment.

Thank you for your consideration of this revised manuscript.

Sincerely,

Ben Raphael, Ph.D.
Professor of Computer Science, Princeton University
Member, Rutgers Cancer Institute of New Jersey
Affiliated Faculty, Herbert and Florence Irving Institute for Cancer Dynamics, Columbia University
Affiliate Member, New York Genome Center

---------------------- REVIEW 1 ---------------------

Upon reviewing the updated manuscript, I find significant improvements in both its readability and accessibility. The changes introduced have made the content more comprehensible and are likely to appeal to a broader audience. I also appreciate that the authors have convincingly addressed all the concerns raised in the initial feedback.

We thank the reviewer for their positive feedback and are pleased to hear that the revised content is both more comprehensible and accessible.

---------------------- REVIEW 2 ---------------------

The paper addresses a critical challenge: inferring tumor phylogenies from copy number profiles, complicated by the inherent property of dependent loci changes. While the CNT model is widely used for copy number evolution, there has been limited development of maximum parsimony methods for it. The author introduces a relaxation of the CNT model and presents the efficient approximation algorithm, Lazac, for small parsimony, complemented by heuristic searching for large parsimony.

The study includes rigorous testing on simulation data, showing that the relaxed ZCNT model consistently exhibits the highest accuracy in inferring copy number phylogenetic trees. Furthermore, the authors conduct a thorough validation on a real breast cancer single-cell dataset, showcasing Lazac's capacity to generate more refined, biologically meaningful, and consistent results, aligned with original clustering labels. Additionally, Lazac consistently produces smaller ZCNT scores compared to the existing tool, Sitka, in most cases, further supported by its better alignment with single-nucleotide variant (SNV) evidence. The qualitative analysis, particularly on sample SA1292, offers a clear demonstration of the method's enhanced reliability.

The paper is well-crafted, with lucid definitions and comprehensive mathematical proofs for the relaxed version of the CNT model. The sections comparing the relaxed version with other methods for solving the original CNT model and analyzing approximation errors provide strong justifications for the theoretical foundation and practical applicability of the approach.
Notably, the authors have diligently addressed previous comments following the paper's earlier submission to RECOMB-CCB, making corresponding edits and enhancing the paper's quality.

We thank the reviewer for their positive comments and suggestions.

Minor comments:

The author used "p" for both copy number profiles and delta profiles in different parts of the paper. It might help make things clearer if they used different variable names.

We agree with the reviewer and have updated the notation to be more clear. Specifically, we now use p for copy number profiles and q for delta profiles, making it easy to distinguish between the two types of profiles.

As for ZCNT with MP performing better than CNT with NJ in all scenarios, is there a plausible explanation for that? Could it be just caused by specific simulation strategy?

This is an important and subtle point. We think that the reason for the observed advantage of ZCNT with maximum parsimony (MP) as compared to CNT with NJ is because the performance of neighbor joining depends upon the correct estimation of the true evolutionary distances between cells. However, the CNT distance is only an approximation for the true evolutionary distance in our simulations.

In cases where the CNT distances exactly match the true evolutionary distances, we expect that this approach will perform similarly or better than ZCNT with MP. Further, we observe that CNT with NJ outperforms ZCNT with NJ in some cases. For example, Supplementary Figure 3 shows that WCND (which, when ran in unweighted mode is CNT + NJ) better recovers the ground truth tree as compared to ZCNT + NJ in terms of Quartet distance, and performs equally as well (or better) in terms of RF distance. To emphasize this point, we have added the following sentence to the main text at the end of Section 7.2:

"However, WCND performed at least as well or strictly better than Lazac-NJ in terms of median RF and Quartet distance for all but two instances (Supplementary Figure 3), emphasizing the importance of accurate estimation of the true evolutionary distance."
